# Multiplex immunofluorescence to measure dynamic changes in tumor-infiltrating lymphocytes and PD-L1 in early-stage breast cancer

**DOI:** 10.1186/s13058-020-01378-4

**Published:** 2021-01-07

**Authors:** Katherine Sanchez, Isaac Kim, Brie Chun, Joanna Pucilowska, William L. Redmond, Walter J. Urba, Maritza Martel, Yaping Wu, Mary Campbell, Zhaoyu Sun, Gary Grunkemeier, Shu Ching Chang, Brady Bernard, David B. Page

**Affiliations:** 1 Earle A. Chiles Research Institute, 4805 N.E. Glisan St., North Tower, Suite 2N87, Portland, OR 97213 USA; 2 Providence Cancer Institute, Portland, OR USA; 3 Department of Pathology, Providence Portland Medical Center, Portland, OR USA; 4 Medical Data Research Center, Providence Health & Services, Portland, OR USA

**Keywords:** Immunotherapy, IRX-2, Multiplex immunofluorescence, Early-stage breast cancer, PD-L1, sTIL, CD8

## Abstract

**Background:**

The H&E stromal tumor-infiltrating lymphocyte (sTIL) score and programmed death ligand 1 (PD-L1) SP142 immunohistochemistry assay are prognostic and predictive in early-stage breast cancer, but are operator-dependent and may have insufficient precision to characterize dynamic changes in sTILs/PD-L1 in the context of clinical research. We illustrate how multiplex immunofluorescence (mIF) combined with statistical modeling can be used to precisely estimate dynamic changes in sTIL score, PD-L1 expression, and other immune variables from a single paraffin-embedded slide, thus enabling comprehensive characterization of activity of novel immunotherapy agents.

**Methods:**

Serial tissue was obtained from a recent clinical trial evaluating loco-regional cytokine delivery as a strategy to promote immune cell infiltration and activation in breast tumors. Pre-treatment biopsies and post-treatment tumor resections were analyzed by mIF (PerkinElmer Vectra) using an antibody panel that characterized tumor cells (cytokeratin-positive), immune cells (CD3, CD8, CD163, FoxP3), and PD-L1 expression. mIF estimates of sTIL score and PD-L1 expression were compared to the H&E/SP142 clinical assays. Hierarchical linear modeling was utilized to compare pre- and post-treatment immune cell expression, account for correlation of time-dependent measurement, variation across high-powered magnification views within each subject, and variation between subjects. Simulation methods (Monte Carlo, bootstrapping) were used to evaluate the impact of model and tissue sample size on statistical power.

**Results:**

mIF estimates of sTIL and PD-L1 expression were strongly correlated with their respective clinical assays (*p* < .001). Hierarchical linear modeling resulted in more precise estimates of treatment-related increases in sTIL, PD-L1, and other metrics such as CD8+ tumor nest infiltration. Statistical precision was dependent on adequate tissue sampling, with at least 15 high-powered fields recommended per specimen. Compared to conventional t-testing of means, hierarchical linear modeling was associated with substantial reductions in enrollment size required (*n* = 25➔*n* = 13) to detect the observed increases in sTIL/PD-L1.

**Conclusion:**

mIF is useful for quantifying treatment-related dynamic changes in sTILs/PD-L1 and is concordant with clinical assays, but with greater precision. Hierarchical linear modeling can mitigate the effects of intratumoral heterogeneity on immune cell count estimations, allowing for more efficient detection of treatment-related pharmocodynamic effects in the context of clinical trials.

**Trial registration:**

NCT02950259.

**Supplementary Information:**

The online version contains supplementary material available at 10.1186/s13058-020-01378-4.

## Background

Immunotherapy with anti-programmed cell death ligand 1 (anti-PD-L1, atezolizumab) was recently approved by the Food and Drug Administration (FDA) for the indication of PD-L1-positive metastatic triple negative breast cancer (TNBC) [1, 2]. However, novel combination immuno-oncology (I-O) therapies will be required to improve efficacy in other therapeutic settings, such as for PD-L1-negative disease, or for less immunogenic breast cancer subtypes such as luminal-type hormone receptor-positive cancers. In an era when numerous I-O agents are being developed clinically [3–5], one promising avenue to accelerate drug development is to develop biomarkers to characterize immune cell (IC) and tumor cell (TC) infiltrates, enabling a comparison of pharmacodynamic effects of various I-O strategies. Here, we describe a methodology that employs multiplex immunofluorescence (mIF) in conjunction with statistical modeling to characterize IC infiltration and PD-L1 expression in the context of early-stage breast cancer (ESBC) I-O clinical trials.

The mIF assay is of particular interest in breast cancer because it may serve to complement two clinically developed I-O biomarkers, PD-L1 expression (by the Ventana SP142 assay), and the hematoxylin and eosin (H&E) stromal tumor-infiltrating lymphocyte (sTIL) score. The SP142 PD-L1 assay is FDA-approved to identify patients with PD-L1^+^ TNBCs who could potentially benefit from the addition of atezolizumab to nab-paclitaxel [1, 6]. The SP142 assay categorizes tumors as PD-L1^+^ if at least 1% of the tumor area is occupied by PD-L1^+^ immune cells (ICs). PD-L1 expression is thought to be dynamic, with biological conditions and/or therapeutic interventions potentially modifying the extent of PD-L1. While the binary designation of PD-L1 status is clinically useful, its ability to serve as a pharmacodynamic biomarker to assess for dynamic PD-L1 change is limited by its semi-quantitative nature and operator dependency. Likewise, the H&E sTIL score uses pathologist estimation of proportion of stromal area occupied by TILs on a single H&E slide as a general gauge of tumor immunogenicity [7]. Similar to PD-L1 testing, sTIL may be clinically useful (as it correlates with survival, chemotherapy response, and potentially immunotherapy response) [8–12]; however, several barriers limit its use as a pharmacodynamic biomarker, including suboptimal inter-observer concordance related to underlying intratumoral heterogeneity of sTILs [13]. Both PD-L1 and sTILs have the limitation of being semi-quantitative assays and require a pathologist to visually estimate ICs, which may sometimes be present in low abundance.

mIF enables estimation of IC counts in high resolution across numerous high-powered magnification fields (hereafter called regions of interest, ROI) and therefore has the potential to produce more accurate and precise estimates of sTILs and PD-L1 expression, relative to the clinical assays. Furthermore, mIF permits more detailed characterization of IC/TC interactions via single-cell quantification of numerous phenotypic surface markers, and spatial localization of cells into various tissue compartments (i.e., tumor versus stroma). Here we use a 6-marker panel of CK, CD3, CD8, FoxP3, CD163, and PD-L1 to visualize, quantify, and phenotype ICs and TCs in ESBC specimens. IC densities and PD-L1 expression are repeatedly sampled across multiple ROIs on a single slide, providing the opportunity to characterize spatial heterogeneity. With appropriate statistical modeling, the repeated sampling of ROIs can be used to improve both accuracy and precision of IC density and PD-L1 expression estimates.

Here, we report mIF data from a phase Ib study of IRX-2, a loco-regional cytokine therapy in early-stage breast cancer (ESBC) [14]. IRX-2 contains various cytokines (including interleukin (IL)-2, IL-1β, interferon-γ, tumor necrosis factor-α, among others) delivered subcutaneously in the distribution of regional lymphatics, and was previously shown to increase tumor lymphocyte infiltration in pre-operative head and neck carcinomas [15–17]. In the phase Ib breast cancer trial, IRX-2 was injected in the peri-areolar tissue (modeled after sentinel lymph node mapping) and was found to be well tolerated, achieving the primary safety endpoint as well as showing evidence of enhanced IC infiltration and lymphocyte activation (measured by RNA sequencing) [14]. Using the paired biopsy and surgical excision specimens from this trial, our objectives of this project were (1) to propose a method for harmonizing mIF with the PD-L1 SP142 and H&E sTIL clinical assays; (2) to illustrate how hierarchical linear modeling can enhance statistical precision of IC density/PD-L1 expression estimates; and (3) to evaluate the influence of ROI sample size on overall statistical power to detect changes in ICs/PD-L1 in the context of a clinical trial.

## Methods

### Parent clinical trial and sample acquisition

Samples were obtained from a pre-surgical phase Ib combination immunotherapy clinical trial (NCT02950259) [14]. The trial is completed, and detailed results have been published, with demographic information summarized in Table 1 [14]. Briefly, patients with ESBC (stage I-III) planned for definitive surgical resection (either lumpectomy or mastectomy) were considered for enrollment at the Providence Cancer Institute (Portland, OR) from May 2016 to May 2018 (*n* = 16). Inclusion criteria comprised any breast cancer subtype, resectable primary tumor > 0.5 cm, Karnofsky Performance Status of ≥ 70%, adequate organ function, absence of steroid-dependent medical conditions, and absence of prior immunotherapy. Diagnostic core biopsies and excisional tumor specimens were collected, processed, and fixed in paraffin (FFPE) per standard-of-care clinical pathology procedures. In this study, a cytokine-based combination immunotherapy approach (IRX-2) was evaluated for feasibility, safety, and immunomodulatory activity (including flow cytometry analysis of lymphocyte subsets, T cell repertoire analysis, and assessment of IC infiltration). Treatment included a combination of single, low-dose cyclophosphamide (300 mg/m^2^ IV) to stimulate antigen presentation and deplete T-regulatory cells, daily oral indomethacin (25 mg three times daily, days 1–21) to modulate myeloid-derived suppressor cells (MDSCs), and locoregional therapy with the investigational agent, IRX-2 (2 mL subcutaneous daily × 10). IRX-2 is a physiologically derived cytokine mixture obtained by ex vivo stimulation (using phytohemagluttinin) of pooled donor peripheral blood leukocytes, from which a stable cytokine mixture is obtained under GMP conditions [18]. The cytokine mixture was delivered subcutaneously adjacent the areola using a method that recapitulated axillary sentinel lymph node mapping, potentially allowing for transmission of cytokines to the tumor-draining lymph node, the putative site of antigen presentation. The study protocol was approved by the Providence Portland Medical Center IRB committee and was conducted in accordance with the ethical standards established by the Declaration of Helsinki.

**Table 1:** Sample summary and clinical results

						Pre-treatment				Post-treatment				Fold change	
ID	TNM	Ki67	Grade	ER/PR	HER2	sTIL H&E (%)	sTIL mIF* (count/pixel)	PD-L1 mIF* (count/pixel)	PD-L1 SP142	sTIL H&E (%)	sTIL mIF* (count/pixel)	PD-L1 mIF* (count/pixel)	PD-L1 SP142	sTIL H&E	PD-L1 SP142
1	pT1c, N1	11%	2	+/+ (100%/100%)	–	8.75	0.48	0.07	IC0	12.5	0.59	0.13	IC0	+ 0.4	0
2	pT2c, pN2	19%	2	+/+ (96%/90%)	–	13.75	1.35	1.04	IC0	20	1.53	0.87	n/a	+ 0.5	n/a
3	pT2, N1	17%	2	+/+ (98%/100%)	–	1.75	0.13	0.07	IC0	1.75	1.25	0.35	IC1	0	+ 1
4	pT2N1	75%	3	−/− (0%/0%)	+	6.25	0.23	0.24	IC1	10	0.74	0.68	IC2	+ 0.6	+ 1
5	pT1c, N1	7%	1	+/+ (98%/92%)	–	3.75	0.88	0.18	IC0	5	0.34	0.09	IC1	+ 0.3	+ 1
6	T2, N1	73%	3	−/− (0%/0%)	+	18.75	0.84	0.01	IC0	18.75	0.94	0.40	IC0	0	0
7	T2, N1	55%	3	+/+ (98%/100%)	–	18.75	1.18	0.54	IC1	20	1.24	0.64	n/a	+ 0.1	n/a
8	pT2, N0	50%	3	+/+ (100%/42%)	–	7.5	1.28	0.97	IC1	16.25	1.68	1.30	IC1	+ 1.2	0
9	pT1b, pN0	11%	2	+/+ (100%/100%)	–	1	0.06	0.06	IC0	5	0.27	0.23	IC0	+ 4	0
10	T1c, N0	33%	2	+/+ (100%/99%)	–	11.5	0.41	0.38	IC1	18.75	1.48	1.33	IC3	+ 0.6	+ 2
11	pT1c, pN1a	38%	3	+/− (69%/0%)	+	6.25	0.35	0.22	IC1	5	1.59	0.65	IC2	−0.2	+ 1
12	pT2, N0	12%	3	+/+ (98%/100%)	+	61.25	2.21	1.81	IC1	72.5	3.52	1.99	IC3	+ 0.2	+ 2
13	pT2, pN0	87%	3	+/+ (100%/7%)	–	31.25	1.69	0.06	IC1	55	1.77	0.98	IC3	+ 0.8	+ 2
14	N/A	N/A	1	+/+ (100%/100%)	–	1	n/a	n/a	n/a	1	n/a	n/a	n/a	0	n/a
15	pT1c, N0	30%	2	+/+ (100%/50%)	–	3.5	1.26	n/a	IC0	7	1.14	0.76	IC2	+ 1	+ 2
16	pT1b, N0	95%	3	−/− (0%/0%)	–	1	0.38	0.03	IC0	13.75	0.99	0.06	IC3	+ 12.8	+ 3

*TNM* stage by the TNM Classification of Malignant Tumors, *Ki67*% of Ki67-positive tumor cells, *ER* estrogen receptor, *PR* progesterone receptor, *HER2* human epidermal growth factor receptor 2, *sTIL* stromal tumor-infiltrating lymphocyte, *H&E* hematoxylin and eosin stain, *PD-L1* programmed death ligand 1, *SP142* Ventana PD-L1 assay, *IC* immune cell; *× 10^−3^

### Stromal TIL scoring by routine hematoxylin and eosin (H&E)

For each treated subject, 5 μm FFPE tissue sections were cut from the core and excision specimens. H&E staining was performed and reviewed by a breast pathologist to confirm the presence of tumor and to evaluate fixation quality. Tissue samples stained by conventional H&E were digitally scanned with Leica SCN400F platform at 20X and maginfication 220x-400x to facilitate blinded evaluation of sTILs using guidelines published by the International Immuno-Oncology Biomarker Working Group on Breast Cancer (i.e., sTILs working group) [7]. The average of sTIL scores of two blinded pathologists were reported as the percentage of stromal area occupied by lymphocytes within areas of invasive carcinoma, excluding areas of carcinoma in situ, necrosis, normal breast/adipose tissue, and biopsy trauma [7, 19].

### PD-L1 positivity by Ventana SP142 assay

Additional 5-μm FFPE slides were stained for clinical PD-L1 scoring using the SP142 assay (Ventana Medical Systems Inc., Tucson, AZ, USA). ICs were scored by two blinded pathologists using published guidelines [6, 20], reported as the proportion of tumor area occupied by PD-L1-staining ICs of any intensity. PD-L1 was scored using the recommended standardized cutoffs (IC0- < 1%, IC1- ≥ 1% to < 5%, IC2 ≥ 5% to 10%, and IC3 ≥ 10%) [6]. In metastatic TNBC, anti-PD-L1 therapy (atezolizumab) is approved in combination with nab-paclitaxel for the treatment of PD-L1 tumors, which corresponds with scores of IC1-IC3 [1, 2]. To serve as internal control for sTIL and PD-L1 score, a contemporary cohort of untreated stage I-III biopsy and matched surgical resection samples (*n* = 14) were analyzed for PD-L1 and sTIL score.

### Multiplex immunofluorescence staining and image acquisition

**Fig. 1:**
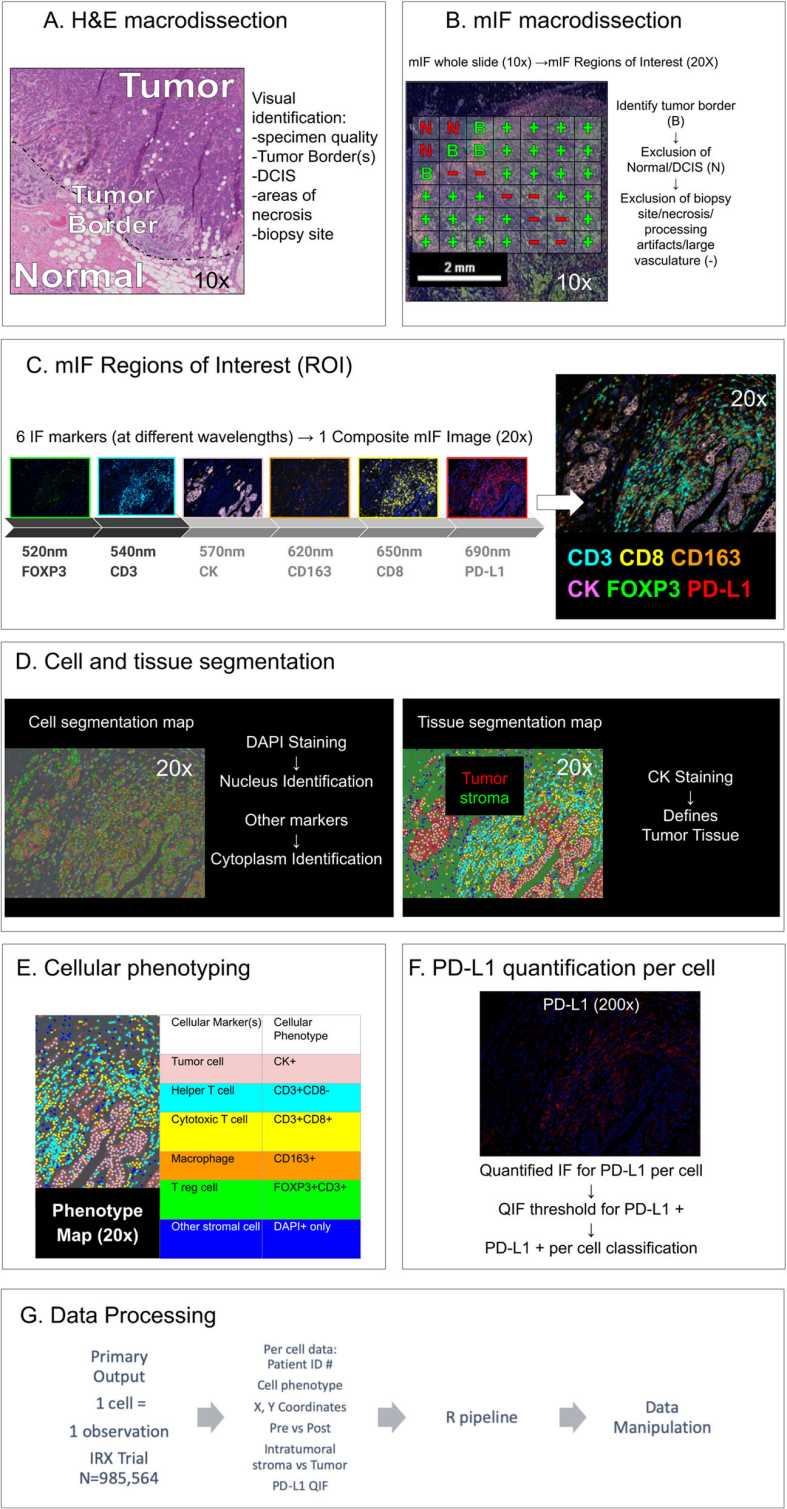
Illustration of mIF workflow and staining. Refer to supplemental figure 2, 3, 4 for example mIF images, validation staining, and examples of QIF levels across phenotypes using the machine-learning based (InForm) phenotyping method. mIF: multiplex immunofluorescence; H&E: hematoxylin and eosin; DCIS: ductal carcinoma in situ; Bx: biopsy; CK: cytokeratin; FOXP3: forkhead box P3; PD-L1: programmed death ligand 1; ROI: region of interest; DAPI: 4′,6-diamidino-2-phenylindole; T reg: T-regulatory; T-S: tumor-stromal; QIF: quantitative immunofluorescence

Additional 5-μm FFPE slides were stained for mIF. Staining methods were validated by the EACRI IHC Core at the Providence Cancer Institute (Portland, OR) and are previously reported [21]. Briefly, sections were deparaffinized and subjected to heat-induced epitope retrieval in Tris-EDTA buffer (pH 9.0). 6-plex panel mIF was performed using the following antibodies: anti-FoxP3 (clone 236A/E7, dilution 1:400, Abcam), anti-PD-L1 (clone E1L3N, dilution 1:1600, Cell Signaling), anti-CD8 (clone SP16, dilution 1:400, Spring Bioscience), anti-CD3 (clone SP7, dilution 1:600, Spring Bioscience), anti-CD163 (clone MRQ26, dilution 1:4, Ventana), anti-CK (clone AE1/AE3, dilution 1:3000, DAKO). Alternative PD-L1 clones are available and are not evaluated here in the context of mIF (SP263, SP142, and 22C3); however, the E1L3N clone was demonstrated to have comparable staining results to these antibodies in a recent clinical validation study [22]. Anti-mouse or rabbit HRP (Biocare Medical) was used as the primary antibody. TSA-conjugated fluorophores (PerkinElmer) were used to visualize each biomarker: Opal 690 (PD-L1), Opal 650 (CD8), Opal 620 (CD163), Opal 570 (CK), Opal 540 (CD3), and Opal 520 (FoxP3). Three percent H_2_O_2_ and microwave treatment in citrate buffer pH 6.0 was performed to prevent cross-reactivity. Tissue slides were incubated with DAPI as counterstain and coverslipped with Prolong antifade mountant (Thermo Fisher Scientific). Whole slides were scanned and digitized at × 10 magnification (PerkinElmer Vectra 3.0) for gross visualization of the tumor, with non-overlapping regions of interest (ROIs) scanned at × 20 (0.36mm^2^) for quantification. ROIs were selected by a study pathologist and included all available tumor-bearing areas containing at least some ICs (> 100 mononuclear cells or more). Regions with empty spaces due to large vasculature, cutting artifacts, or other artifacts were avoided. The number of ROIs per slide ranged from 8 to 32 (mean 16). The workflow is graphically depicted in Fig. 1, and additional details on method are furnished upon request.

### Data analysis

InForm software (PerkinElmer, package 2.4) was used to segment tumor regions (stroma versus tumor) and phenotype cells based upon marker expression. Technical details on the method [23], step-by-step instructions [24], and examples of application in other ESBC datasets are published [25]. Four representative ROIs for each specimen were used for training and capturing the heterogeneity of staining. The process can be summarized in four steps. First, digital images were processed and converted to data matrices according to optical density. Second, the ROIs were segmented into tumor and stromal compartments, which requires a manual step of selecting several small representative areas (containing 3–15 nuclei) for each compartment (using H&E as a benchmark). InForm uses these selections to train and segment the remaining regions, which were then manually compared with H&E for accuracy. Third, cells were labeled according to the most likely phenotype using an Inform-based machine-learning algorithm guided by manual selection of several cells per phenotype of interest. In this experiment, cells were categorized according to the following phenotypes: tumor cells (CK^+^), helper T cells (CD3^+^CD8^−^), cytotoxic T cells (CD3^+^CD8^+^), regulatory T cells (T reg, CD3^+^FoxP3^+^), macrophages (CD163^+^), and other stromal cells (DAPI+ only). PD-L1 expression was analyzed as both a continuous variable (reporting mean quantitative immunofluorescence [QIF] intensity for each cell), as well as a binary PD-L1+/− phenotype (described in the “Results” section). Next, image reports and phenotype maps were generated for each ROI. Finally, output files were generated containing per-cell observations with the following features: patient identification number, sample type (pre-treatment biopsy versus post-treatment excision), ROI unique identifier, cellular phenotype, tissue compartment (tumor versus stroma) and areas for each compartment, Cartesian coordinates (*x-* and *y*-axes), and mean cellular PD-L1 QIF intensity (illustrated in Fig. 1).

### Statistical methods

An important goal was to evaluate a statistical approach that could account for heterogeneity within different areas of the tumor, thus improving reliability of estimating overall IC count and PD-L1 expression. A Poisson generalized linear mixed model (GLMM) was used with a log-linear effect of prevalence, an offset of log (area) to make the expected number of cells proportional to the area, using the function “glmer” in R package “lme4.” This well-described model [26, 27] accounts for differences in stromal/tumor area within each ROI, correlation of time-dependent measurements, and variation among patients and ROIs when estimating the relative influence of treatment exposure on IC density or PD-L1 expression. The model was selected as the best fit by likelihood ratio test, AIC and BIC criterion based on the observed data. The formal formula of the model can be written as log (*λ*_*ij*_) = offset (log (Area)) + *β*_0_ + *β*_1_ *T*_*i*_ + *b*_*i*_ + *b*_*j*(*i*),_ where *λ*_*ij* =_ *E* (*y*_*ij*_) is the mean of cell count, *y*_*ij*_ (e.g., CD3 cell counts) in the *j*th ROI of the *i*th subject, with *T*_*i*_ being a binary variable indicating post- versus pre-treatment, *b*_*i*_ and *b*_*j*(*i*)_ representing random effects pertaining to *i*th subject and *j*th ROI. In the model, counts are adjusted for the compartment area. Treatment-associated effects (i.e., fold change [FC] in density from pre- to post-treatment) with 95% confidence intervals were estimated by exponentiation of the coefficient for post-treatment.

## Results

### Evaluation of TILs by mIF

**Fig. 2:**
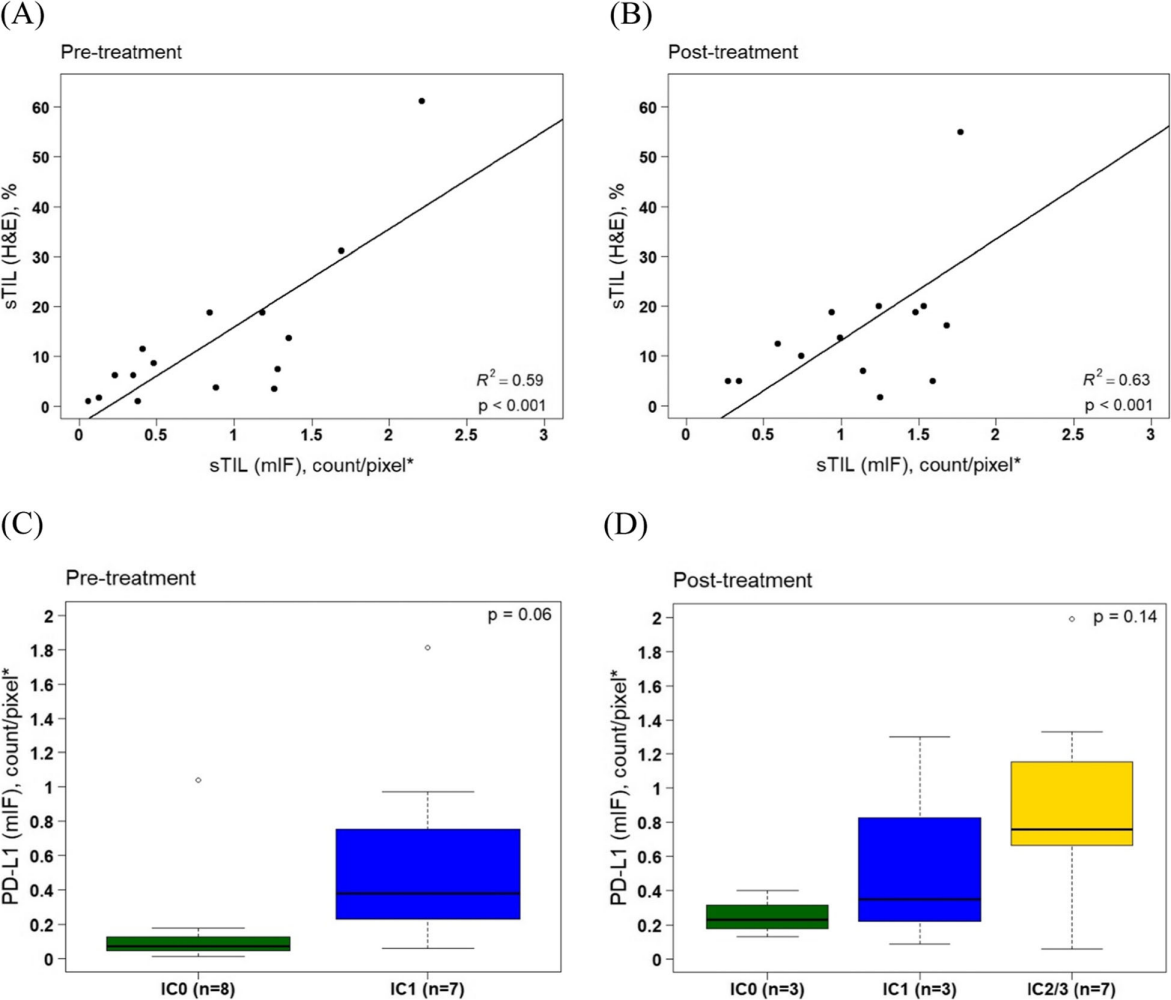
Harmonization of clinical sTIL and PD-L1 assays with mIF. Correlation scatterplot of sTIL (H&E) and sTIL (mIF) for pre-treatment (**a**) and post-treatment (**b**) with fitted linear regression lines. Mean PD-L1 mIF score comparison between PD-L1 SP142 category (IC 0, IC1, IC2/3) for pre-treatment (**c**) and post-treatment (**d**) using Jonckheere-Terpstra test. **s**TIL: stromal tumor-infiltrating lymphocyte; mIF: multiplex immunofluorescence; IC: immune cell; PD-L1: programmed death ligand 1

IRX-2 was previously shown to increase the mean H&E sTIL score, upregulate PD-L1 expression, and upregulate immune-related gene signatures [14]. We sought to develop a method of measuring sTILs using mIF that would closely recapitulate the H&E sTIL score (sTIL_H&E_), but potentially with greater precision compared to the semi-quantitative clinical assays. The sTILs working group guidelines define sTILs as all stromal mononuclear cells, which include lymphocytes and plasma cells but exclude other ICs such as macrophages, with the score being a percentage of stromal area occupied by these cells [7, 19]. To recapitulate this, we defined the sTIL_mIF_ score as the sum of counts of all stromal T cells (helper, cytotoxic, and regulatory) divided by stromal area (Table 2). Plasma cells are not captured by this panel and may be present in ESBC, but are usually clustered in sparse ROIs [28]. Results are illustrated in Table 1 and Fig. 2a, b. sTIL_H&E_ and sample mean sTIL_mIF_ scores (defined as mean sTIL_mIF_ across all ROI) were correlated for both pre-treatment (*r*^2^ = 0.59, *p* < .001) and excisional samples (*r*^2^ = 0.63, *p* < .001). sTIL_mIF_ scores varied substantially across ROIs within a sample, with a mean coefficient of variation (CV) of 0.61 (range 0.27–1.44), indicative of intra-sample heterogeneity.

**Table 2:** Summary of metrics and definitions

Metric	Notation	Formula	Analysis level (cell, ROI, slide)	Attributes
**Count/density metrics**				
sTIL score	sTIL_H & E_	% of stromal area occupied by TILs on one H&E slide	Slide	Generalized metric for presence/absence of immune cells, prognostic in breast cancer and predictive of chemotherapy benefit and potentially immunotherapy benefit (references [10, 11])
mIF sTIL score	sTIL_mIF_	$\frac{{\mathrm{Count}}_{\mathrm{sCD}3+/\mathrm{sCD}8+/\mathrm{sFOXP}3}}{{\mathrm{area}}_s}$	ROI	Quantification of therapy-related changes in overall TILs. Analogous to the H&E sTIL score and therefore may have potential as a prognostic/predictive marker
Cell count	Count_CD8_	#CD8^+^ ICs within ROI	ROI	
Cell density	Density_sCD8_	$\frac{{\mathrm{Count}}_{\mathrm{sCD}8}}{{\mathrm{Area}}_s}$	ROI	May be a more reliable measurement of IC quantity because it adjusts for differences in stroma/tumor compartmentalization across tumor regions
**PD-L1 metrics**				
SP142 PD-L1 score	PDL1_SP142_	% of tumor area occupied by PDL1-positive ICs	Slide	Categorical metric for ascertaining IC PD-L1 positivity, predictive of immunotherapy response (anti-PD-L1) in triple negative breast cancer
PD-L1 cell intensity	QIF_CD8_	PDL1 mean whole cell QIF	Cell	Quantification of PD-L1 intensity on individual cells may be useful for evaluating effect of therapies on dynamic PD-L1 expression on tumor or ICs
mIF PD-L1 score	PDL1_mIF_	${\mathrm{Count}}_{\mathrm{PDL}{1}^{+}\ \mathrm{IC}}$ (CD3^+^ or CD163^+^ & QIF > 2.6)	ROI	Quantification of overall number of PD-L1+ ICs. Analogous to the SP142 PDL1 score and therefore may have potential as a predictive marker

*ROI* region of interest, *mIF* multispectral immunofluorescence, *sTIL* stromal tumor-infiltrating lymphocyte, *H&E* hematoxylin and eosin, *TILs* tumor-infiltrating lymphocytes; *s* stromal, *IC* immune cell, *SP142* Ventana SP142 PD-L1 assay, *PD-L1* programmed death ligand 1, *QIF* quantitative immunofluorescence

**Fig. 3:**
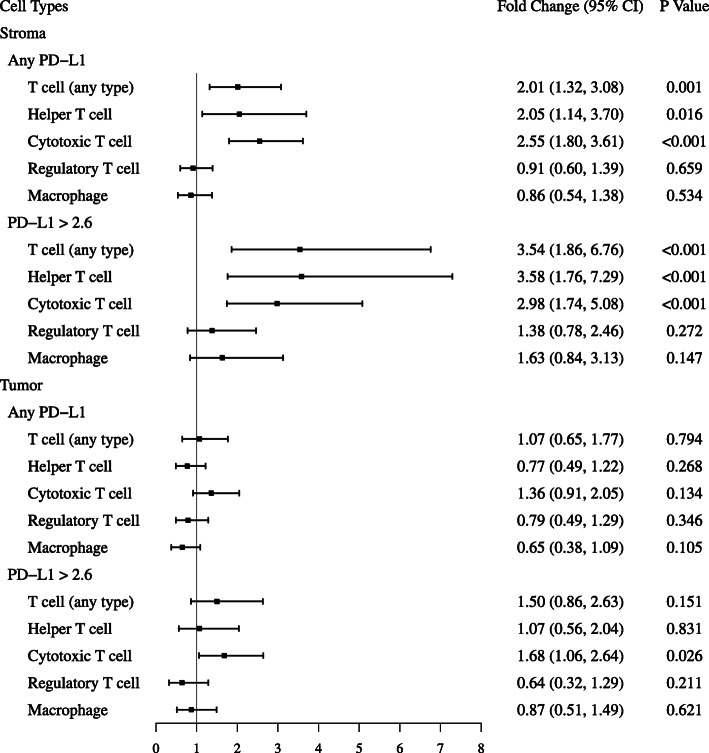
Forest plot of IRX-2 immunotherapy effects on lymphocyte subsets. PD-L1: programmed death ligand 1; CI: confidence interval

mIF allows for evaluation of treatment-related effects on specific cell lineages across both stromal and tumor compartments. Table 3 and Fig. 3 illustrate the effects of IRX-2 on stromal and tumor cell density observed across various lineages. We evaluated therapy-related fold changes in cell count using the paired *t*-test versus adjusted counts using the hierarchical linear model. The adjusted estimates of fold change using the hierarchical linear model tended to be higher and with smaller *p* values, relative to estimates of fold change using the paired *t*-test. IRX-2 was associated with significant increases in stromal cytotoxic T cells and helper T cells, but no change in regulatory T cells or macrophages. Within the tumor compartment, therapy was associated with increases in cytotoxic T cells; however, this only achieved significance using the hierarchical modeling approach. We also evaluated cellular ratios to evaluate for therapy-related shifts in IC phenotype predominance (supplemental table 1) and identified a reduction in the macrophage/T cell ratio (median FC − 0.56, mean FC −.091; range − 0.99 to + 3), and the regulatory T cell /cytotoxic T cell ratio (median FC − 0.78; mean FC − 0.58; range − 0.90 to + 0.75).

**Table 3:** IRX-related effects on various immune cell subpopulations within stromal and tumor tissue compartments

	Summary statistics		Unadjusted^1^		Adjusted^2^	
	Pre-IRX, mean density (SD)* (count/pixel)	Post-IRX, mean density (SD)* (count/pixel)	Fold change of mean density (95% CI)	*p* value	Fold change of mean density (95% CI)	*p* value
**Stroma**						
**Any PD-L1**						
T cell (any type)	0.85 (0.63)	1.27 (0.78)	1.8 (1.14 2.84)	**0.015**	2.01 (1.32 3.08)	**0.001**
Helper T cell	0.56 (0.47)	0.79 (0.59)	1.84 (0.97 3.48)	0.060	2.05 (1.14 3.70)	**0.016**
Cytotoxic T cell	0.22 (0.19)	0.42 (0.30)	2.18 (1.47 3.23)	**< 0.001**	2.55 (1.80 3.61)	**< 0.001**
Regulatory T cell	0.07 (0.05)	0.07 (0.06)	0.92 (0.57 1.49)	0.729	0.91 (0.60 1.39)	0.659
Macrophage	0.24 (0.18)	0.19 (0.12)	0.86 (0.54 1.37)	0.498	0.86 (0.54 1.38)	0.534
**PD-L1-positive†**						
T cell (any type)	0.40 (0.57)	0.7 (0.59)	3.04 (1.48 6.23)	**0.005**	3.54 (1.86 6.76)	**< 0.001**
Helper T cell	0.28 (0.44)	0.47 (0.46)	3.31 (1.45 7.57)	**0.008**	3.58 (1.76 7.29)	**< 0.001**
Cytotoxic T cell	0.08 (0.11)	0.19 (0.21)	2.44 (1.28 4.63)	**0.011**	2.98 (1.74 5.08)	**< 0.001**
Regulatory T cell	0.05 (0.05)	0.05 (0.05)	1.39 (0.71 2.73)	0.311	1.38 (0.78 2.46)	0.272
Macrophage	0.12 (0.13)	0.14 (0.11)	1.66 (0.75 3.68)	0.192	1.63 (0.84 3.13)	0.147
**Tumor**						
**Any PD-L1**						
T cell (any type)	0.16 (0.17)	0.15 (0.15)	1.04 (0.61 1.8)	0.865	1.07 (0.65 1.77)	0.794
Helper T cell	0.10 (0.11)	0.07 (0.07)	0.81 (0.52 1.24)	0.304	0.77 (0.49 1.22)	0.268
Cytotoxic T cell	0.06 (0.05)	0.08 (0.09)	1.26 (0.81 1.94)	0.282	1.36 (0.91 2.05)	0.134
Regulatory T cell	0.02 (0.01)	0.02 (0.01)	0.72 (0.41 1.28)	0.242	0.79 (0.49 1.29)	0.346
Macrophage	0.04 (0.05)	0.03 (0.02)	0.71 (0.37 1.37)	0.282	0.65 (0.38 1.09)	0.105
**PD-L1-positive†**						
T cell (any type)	0.11 (0.17)	0.09 (0.09)	1.42 (0.71 2.87)	0.295	1.50 (0.86 2.63)	0.151
Helper T cell	0.08 (0.13)	0.05 (0.05)	0.98 (0.51 1.91)	0.955	1.07 (0.56 2.04)	0.831
Cytotoxic T cell	0.04 (0.04)	0.05 (0.05)	1.63 (0.80 3.31)	0.155	1.68 (1.06 2.64)	**0.026**
Regulatory T cell	0.03 (0.05)	0.01 (0.01)	1.00 (0.49 2.05)	0.999	0.64 (0.32 1.29)	0.211
Macrophage	0.03 (0.05)	0.02 (0.02)	0.84 (0.51 1.39)	0.463	0.87 (0.51 1.49)	0.621
**Stroma and tumor**						
**PD-L1-positive†**						
PD-L1+ IC^3^	0.41 (0.53)	0.70 (0.54)	2.75 (1.36 5.53)	**0.008**	3.14 (1.68 5.87)	**< 0.001**

^1^Paired *t*-test based on log-transformed density

^2^Poisson Generalized Linear Mixed-Effects Model (GLMM), with a log-linear effect of prevalence, an offset of log (area) (to make the expected number of cells proportional to area), the fixed effect of time (pre vs post), and two random effects of patients and ROI, to account for the variation in patients and ROI

*× 10^− 3^; †PD-L1 > 2.6

T cell (any type) = combination of CD3+, CD8+, and FOXP3+ cell types; helper T cell = CD3+; cytotoxic T cell = CD8+; regulatory T cell = FOXP3+; macrophage = CD163

*PD-L1* programmed death ligand 1, *CI* confidence interval

### Evaluation of PD-L1 expression by mIF

IRX-2 is associated with increases in PD-L1 expression using the SP142 clinical assay (PDL1_SP142_) relative to untreated controls (Table 1). We sought to develop a method of measuring PD-L1 using mIF (PDL1_mIF_) that would closely recapitulate the PDL1_SP142_ score. By SP142, pathologists visually classify individual ICs as PD-L1 positive versus negative, then estimate the total tumor area occupied by PD-L1-positive cells. To recapitulate this method by mIF, an accurate per-cell QIF cutoff for PD-L1 positivity would be necessary. We used InForm software to create simulated chromogenic PD-L1 images derived from individual ROIs of various mIF samples. With these images, the study pathologist was asked to classify 231 randomly selected ICs of various PD-L1 QIF across 4 specimens, and with these classifications, a receiver operating characteristic (ROC) curve was generated to determine the most accurate QIF cutoff for per-cell PD-L1 positivity. The optimal QIF level was 2.6, corresponding with an AUC of 0.97, sensitivity of 91%, specificity of 99%, and classification accuracy of 95% (supplemental figure 1). Using the 2.6 cutoff, accuracy for individual specimens ranged from 92 to 100% (mean 96%), suggesting that a QIF cutoff of 2.6 would be adequate across tumor samples. We defined the PDL1_mIF_ score as the count of all ICs within an ROI with QIF > 2.6. As illustrated in Table 3 and Fig. 2c, d, we demonstrate a mean 3.14-fold increase of PDL1_mIF_ IC density related to treatment. PDL1_mIF_ scores were correlated with the PDL1_SP142_ assay, with average densities increasing concordantly according to SP142 IC category (Fig. 2c, d). In a mixed effects model that accounts for correlations across pre/post-treatment samples, PDL1_mIF_ for IC1 tumors was 2.99-fold higher (*p* = .04) than IC0 tumors, and PDL1_mIF_ for IC2/3 tumors was 5.95-fold higher (*p* = .003) than IC0 tumors. Similar to sTIL_mIF_ scores, PDL1_mIF_ scores ranged widely across ROI within samples (CV 0.78, range 0.3–1.29), highlighting the importance of adequate ROI sampling to characterize tumors.

### Power analyses

**Fig. 4:**
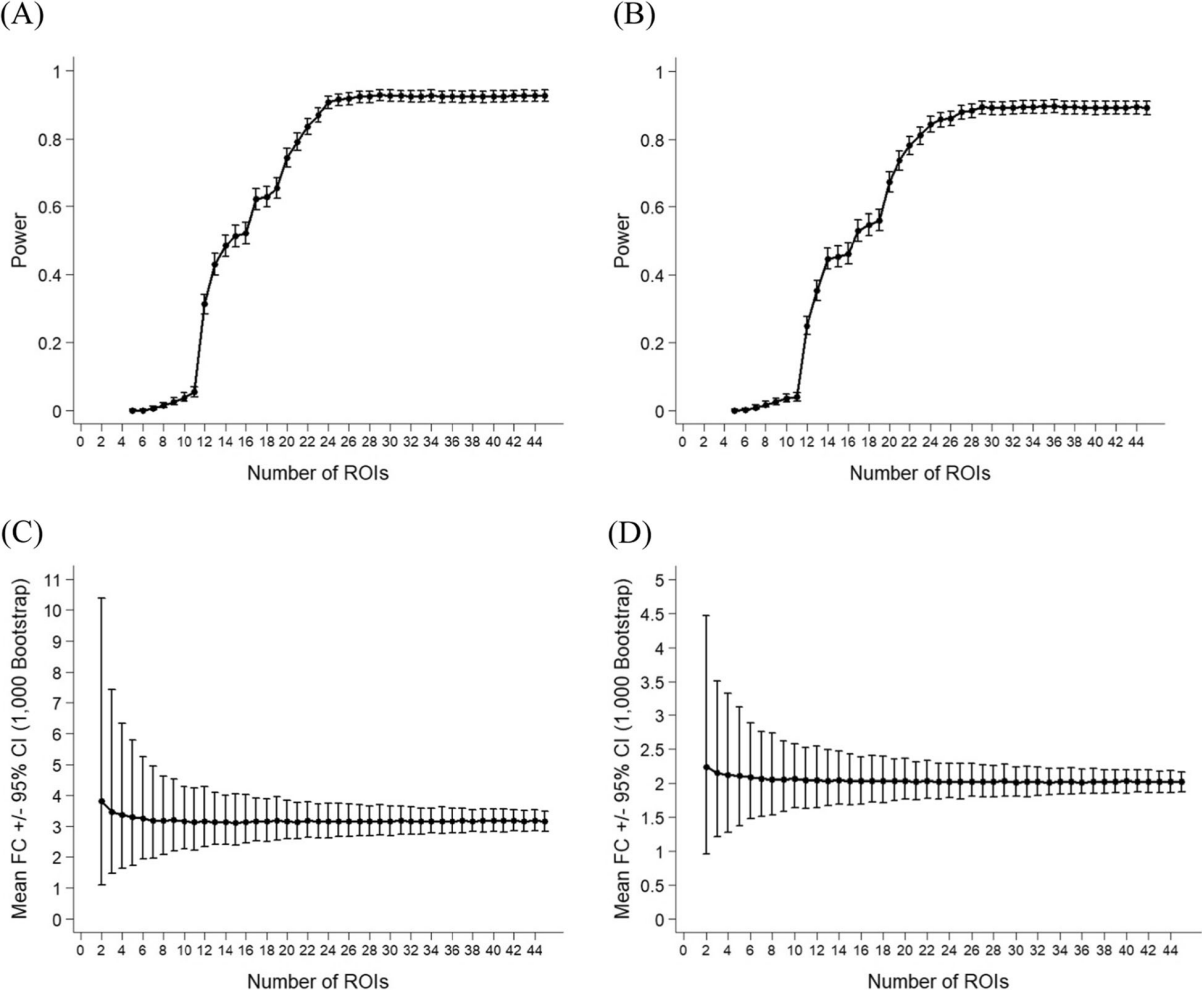
Power analyses illustrating the effects of ROI sample size. **a**, **b** Power curve according to ROI size, holding patient sample size (*n* = 15) fixed, for the PD-L1 mIF biomarker (**a**) and the sTIL mIF biomarker (**b**) produced using a Monte Carlo simulation approach (*n* = 1000 simulations), based on the generalized linear mixed effects model and the observed data structure [i.e., effect sizes and variations obtained post hoc from the pilot experiment]. **c**, **d** Mean fold change and 95% confidence intervals based upon a bootstrap simulation method (1000 simulations) according to ROI size, for the PD-L1 mIF biomarker (**c**) and the sTIL mIF biomarker (**d**). ROI: region of interest; FC: fold change; CI: confidence interval

Because of the computational labor associated with mIF, it is of interest to ascertain how many ROIs must be analyzed to adequately represent the entirety of the specimen. We evaluated whether fewer ROIs would be sufficient to detect treatment-related change in a clinical trial, using PD-L1_mIF_ and sTIL_mIF_ scores as examples. Holding patient sample size (*n* = 15) fixed, a Monte Carlo simulation approach (*n* = 1000 simulations) was employed to calculate power across various ROI sample sizes, based on the Poisson hierarchical linear model described in section “Statistical methods”, and the observed data structure [i.e., effect sizes and variations obtained post hoc from a pilot experiment] using the “*simr*” package in R [29]. The analyses show that with 15 subjects, on average, 22 ROIs within each subject would be required to detect the treatment effect (FC = 3.14, 95% CI = 1.68–5.87) for PD-L1_mIF_ (Fig. 4a), and 24 ROIs within each subject would be required to detect the treatment effect (FC = 2.01, 95% CI = 1.32–3.08) for sTIL_mIF_ (Fig. 4b), to attain at least 80% power at a significance level of 0.05. A reduction in ROI sampling led to a substantial decrease in statistical power to detect changes in both PD-L1_mIF_ and sTIL_mIF_ (Fig. 4a, b), likely related to the high degree of variation in PD-L1_mIF_ and sTIL_mIF_ across ROIs within the same specimen. In our experience, the range of evaluable ROIs per specimen was 8–32 (mean 16), and therefore in the context of similar trials, it would be advisable to evaluate as many ROIs as possible on one slide to detect effects similar to described. In studies evaluating smaller effect sizes, greater sample sizes would be required.

We compared the hierarchical linear model with more conventional methods of reporting treatment-related change in clinical trials. The most common conventional method is to test fold changes in means using the paired *t*-test. Using the above Monte Carlo method for the hierarchical linear model, holding per-specimen ROI fixed based on observed data structure, we estimated that *n* = 11 patients would be required to detect a 3.14-fold change in PD-L1_mIF_, and *n* = 13 patients would be required to detect a 2.01-fold change in sTIL_mIF_ with 80% power at a significance level of 0.05 (supplementary table 2). However, by the paired *t*-test approach [30], a sample size of *n* = 13 would be required to detect similar changes in PD-L1_mIF_ (FC = 2.75, CV = 1.08), whereas a sample size of *n* = 25 would be required to detect similar changes in sTIL_mIF_ (FC = 1.80, CV = 0.83). These data suggest that the hierarchical linear model is associated with increased statistical power, or reductions in required subject enrollment size, in the context of comparative trials.

Finally, we evaluated the potential impact of ROI sample size on estimation of treatment-related changes in sTILs or PD-L1 expression. Using a bootstrap method assuming a clinical trial with *n* = 15 patients, fold change estimates of sTIL and PD-L1 scores were simulated 1000 times across various ROI sizes to create a mean and 95% CI of estimated FC. As illustrated in Fig. 4c, d, ROI sample sizes of < 10 were associated with wide CIs, whereas ROI sample sizes of > 15 were sufficient to optimize accuracy.

## Discussion

Innumerable I-O strategies show promise in preclinical breast cancer models either as monotherapy or in combination with approved therapies (T cell agonists, trastuzumab, chemotherapy, radiotherapy, or targeted therapy) [1–4, 31–33]. Pre-operative I-O clinical trials in ESBC provide the opportunity to efficiently compare pharmacodynamic activity using serial tissue-based comparative biomarkers, while also providing pathologic outcomes as a meaningful surrogate of disease-free recurrence [34]. mIF has been proposed as a promising biomarker, as it has been shown to be concordant with clinical PD-L1 assays in ESBC [35], and in a recent meta-analysis outperformed clinical PD-L1 testing, quantification of tumor mutational burden, or gene expression profiling in predicting immunotherapy response [36]. Here, we provide additional guidance on how mIF can be used as a pharmacodynamic biomarker in the context of ESBC I-O pre-operative clinical trials. We show that mIF estimates of PD-L1 expression and sTIL/IC density correlate with the validated clinical assays, but with higher resolution to measure treatment-related pharmacodynamic changes. It has recently been suggested that both PD-L1 and sTIL clinical assays be co-analyzed to enhance predictive/prognostic performance [37]. As illustrated in this manuscript, mIF provides granular detail on single-cell PD-L1 expression across cellular phenotypes, which can be used to categorize tumors based upon ratios of PD-L1-expressing cells, phenotypic predominance patterns of PD-L1+ cells (i.e., macrophage v. lymphocyte), and spatial patterning of PD-L1. As a future direction of investigation, we propose that clinical investigators prioritize the inclusion of mIF in clinical trials in tandem with the clinical assays, so the unique predictive/prognostic utility of these added data can be adequately interrogated.

We identified several aspects of mIF that might be useful in addressing the pitfalls of clinical H&E sTIL assessment, which were recently summarized from the RING studies [13]. First, by H&E, it was found that non-lymphocyte cells or intraepithelial TILs could be misclassified for sTILs by pathologists. mIF could substantially mitigate this source of error, by employing multiple cell surface markers to accurately classify lymphocytes. Second, it was found that pathologists exhibited different set-points/scales for quantifying sTILs by H&E, resulting in substantial inter-observer discordance. This pitfall could be in the future be mitigated by mIF once the staining, imaging, and analysis workflow becomes harmonized across institutions. Efforts are ongoing via the National Institutes of Health Cancer Immune Monitoring and Analysis Centers (CIMAC) to standardize and validate a mIF workflow [38]. A third source of error was heterogeneity of sTIL counts across areas of the tumor. One proposed solution to mitigate this error is to sample and average sTIL counts across multiple ROIs [13]. Using mIF, it is feasible to estimate sTIL counts across a large number of ROIs, and we demonstrate that adequate ROI sampling is important for stabilizing estimates of treatment-related changes in sTILs in the context of clinical trials.

Statistical modeling has been underexplored as a method for improving accuracy and precision of sTIL/IC density estimation. To date, there is no universally adopted approach for the statistical treatment of mIF output data. By convention, many investigators collapse ROI IC density estimates into a mean per-sample score, which does not fully utilize the added information derived from repeated sampling across ROIs. As an alternative, we demonstrate statistical modeling can improve statistical power and minimize potential detrimental confounding effects of intratumoral heterogeneity. As illustrated in Table 3, statistical modeling was associated with a narrowing of confidence intervals of IC estimates, and smaller observed *p* values. Furthermore, compared to conventional *t*-testing of means, the hierarchical linear modeling method reduced the required patient enrollment size from *n* = 25 to *n* = 13 to show an effect of IRX-2 on sTILs.

We also illustrate how mIF can be also used to evaluate more complex hypotheses related to I-O treatment effect. For example, based upon preclinical models and previous trials data, it was hypothesized that locoregional cytokine perfusion (IRX-2) would increase lymphocyte trafficking and facilitate PD-L1 upregulation within the breast tumor via modulation of the JAK-STAT pathway [17]. Using mIF, we confirmed that IRX-2 is associated with increases in sTILs and PD-L1 upregulation in the tumor microenvironment, as well as a shift in the ratio of cytotoxic T cells to CD163+ macrophages and regulatory T cells. These findings are corroborated by previously published data from gene expression profiling, clinical SP142/H&E sTIL assays, and T cell receptor DNA sequencing [14]. Based upon these encouraging findings, we are conducting a trial to compare single-dose anti-PD-1 +/− IRX-2 (*n* = 15 per arm) as an induction therapy to potentially enhance immune infiltration prior to neoadjuvant chemotherapy plus pembrolizumab in stage II-III TNBC (NCT04373031). In the future, the spatial output data derived from mIF can be used to evaluate spatial hypotheses, such as whether cytokine therapy permits aggregation or penetration of tumors into the tumor/stromal interface. Such a hypothesis could be evaluated by comparing pre versus post-treatment densities of buffer zones surrounding the tumor/stromal interface.

Our approach is not without limitations. First, because the assay is limited to 7 markers, B cell markers were not included, and this may have influenced the overall estimation of sTILs (since B cells would be included in the H&E sTIL score). It is possible to customize mIF with different markers; however, careful attention must be paid to ensure that each panel is properly validated using ESBC specimens, and therefore for this pilot study, we opted to use a previously validated panel for which we have extensive experience and publication [21, 39]. Future improvements in technology are anticipated to allow for simultaneous measurement of additional markers. A second limitation is the lack of a treatment control, which precluded assessment of potential confounding effects of time and/or biopsy trauma. This will be addressed in the ongoing randomized phase II trial. A third limitation is the resource-intensive nature of our approach, which requires acquisition and analysis of all lymphocyte-bearing ROIs in a given sample. This process may require 24 h of processing or greater per specimen; however, we illustrate that the efforts are worthwhile in the context of clinical research as they may reduce sample size requirements. In clinical trials, per-patient expenses, time, and effort are likely to far outweigh the added time and cost required to sample more ROIs. Future advances in technology may permit more rapid acquisition and analysis of whole-slide data, for example using the PerkinElmer Polaris system, which is being validated by our group and others. The fourth limitation is that breast cancer subtypes and/or clinical settings may have unique histologic and immunologic features and therefore our power calculations may not be externally valid in other settings. For example, baseline sTIL levels and PD-L1 expression are lower in hormone-sensitive breast cancers relative to TNBC, and therefore when designing a clinical trial, the power analyses would have to be repeated or modified to account for these expected differences in baseline.

As a future direction, the described statistical modeling can be amended to incorporate data on spatial locations of each ROI and/or each cell, which has further potential to improve estimation. For example, because IC densities of immediately adjacent ROIs may be correlated, the accuracy of the model could be improved after adjustment for spatial autocorrelation. Similarly, topographical features such as leading invasive margin of the tumor are expected to influence IC densities and may be accounted for in the model [13]. We are piloting advanced spatial modeling that would enable adjustment of IC densities according to spatial distance from observed topographical landmarks, as well as more advanced methods to exclude non-interpretable areas within ROI to improve accuracy.

## Conclusion

mIF may be used in the context of ESBC I-O trials to detect dynamic changes in ICs and PD-L1 expression associated with treatment. Our mIF method is concordant with H&E sTIL and SP142 PD-L1 clinical assays, but enhances precision of these measurements by accounting for intratumoral heterogeneity. The statistical modeling approach could also be evaluated as a method of improving performance of the H&E sTIL clinical assay. The method may also be used to evaluate other features of the immune response, such as treatment-related changes in cellular ratios, or immune cell clustering patterns. Our approach requires validation, which is planned using specimens from a phase II neoadjuvant phase II trial of pembrolizumab +/− IRX-2. (NCT04373031). The method also has promise to be explored as a predictive biomarker in the context of neoadjuvant anti-PD-1/L1 plus chemotherapy, as PD-L1 expression alone did not predict benefit in this setting [40].

## Supplementary Information

**Additional file 1: Table S1.** Estimated cell count ratios. Table S2. Power analysis using Monte Carlo simulation approach (*n* = 1000 simulations), based on the generalized linear mixed-effects model and the observed data structure [i.e., effect sizes and variations obtained post – hoc from a pilot experiment]. Table S3. PD-L1 and sTIL scores for individual patients with coefficients of variation.

**Additional file 2: Figure S1.** Receiver operating characteristic curve for determining PD-L1 threshold. (A) Example images of high powered (20x) ROIs, InForm pathology view (showing only PD-L1 expression by mIF) counterstained with DAPI. Green is used here to label random cells that could be visually classified as PD-L1 positive versus negative by the reading pathologist, and used to ascertain an appropriate QIF cutoff for PD-L1 positivity. (B) Histogram of the distribution average per-cell QIF PD-L1 levels of 55,108 cells pooled from 24 ROI across 4 patients). (C) ROC curve illustrating sensitivity and specificity for given PD-L1 QIF thresholds. A threshold of ≥2.6 was selected, corresponding with sensitivity of 91%, specificity of 99%, AUC of 0.97, and accuracy of 95%. ROI: region of interest; PD-L1: programmed death ligand 1; mIF: multispectral immunofluorescence; DAPI: 4′,6-diamidino-2-phenylindole; QIF: quantitative immunofluorescence; ROC: receiver operating characteristic; AUC: area under the curve.

**Additional file 3: Figure S2.** Example of mIF staining and illustration of QIF levels across phenotypes using machine-learning based (InForm) phenotyping method. PD-L1: programmed death ligand 1; DAPI: 4′,6-diamidino-2-phenylindole; CK: cytokeratin; FOXP3. (A) mIF images showing CK+ tumor nests with CD8 expression (red), CD3 expression (green), and yellow indicating co-expression; (B) expected expression patterns of helper T cells, cytotoxic T cells, and regulatory T cells; (C-D) Mean quantitative immunofluorescence of CD3 and CD8 for each of the phenotypes; (E-G) Comparison of mean QIF expression patterns for various phenotypes macrophage CD163 expression to other cells. CK: cytokeratin; FOXP3: forkhead box P3; reg: regulatory; QIF: quantitative immunofluorescence.

**Additional file 4: Figure S3.** mIF antibody validation. Antibody validation was performed using conventional/standard chromogenic stain on human FFPE tonsil tissue. TSA-Opal fluorescent stain was performed on the adjacent slides. Images were acquired with a Vectra 3 Automated Quantitative Pathology Imaging system. FOXP3: forkhead box P3; CK: cytokeratin; PD-L1: programmed death ligand 1.

**Additional file 5: Figure S4.** mIF antibody validation, PD-L1 staining. Antibody validation (aPD-L1; clone E1L3N) was performed using conventional/standard chromogenic stain on human FFPE placenta tissue. TSA-Opal fluorescent stain was performed on the adjacent slides. Images were taken with a Vectra 3 Automated Quantitative Pathology Imaging system. PD-L1: programmed death ligand 1; H&E: Hematoxylin and eosin.

## Data Availability

The full mIF datasets will be furnished upon request at a later date after upon completion of remaining analyses and publication.

## Abbreviations

AUC: Area under the curve
CI: Confidence interval
CK: Cytokeratin
CV: Coefficient of variation
ESBC: Early-stage breast cancer
FC: Fold change
FDA: Food and Drug Association
FFPE: Formalin fixed paraffin embedded
GLMM: Generalized linear mixed model
H&E: Hematoxylin and eosin
I-O: Immuno-oncology
IC: Immune cell
IRB: Institutional review board
MDSC: Myeloid-derived suppressor cell
mIF: Multiplex immunofluorescence
PD-L1: Programmed death ligand 1
QIF: Quantitative immunofluorescence
ROI: Region of interest
sTIL: Stromal tumor-infiltrating lymphocyte
TC: Tumor cell
TNBC: Triple negative breast cancer
