# Supplementary material for: Multiplex immunofluorescence to measure dynamic changes in tumor-infiltrating lymphocytes and PD-L1 in early-stage breast cancer
Source: Breast Cancer Res. 2021 Jan 7;23:2. doi: 10.1186/s13058-020-01378-4 (PMC7788790; doi:10.1186/s13058-020-01378-4)
Supplement: Supplementary file 1 — Additional file 1: Table S1. Estimated cell count ratios. Table S2. Power analysis using Monte Carlo simulation approach (n = 1000 simulations), based on the generalized linear mixed-effects model and the observed data structure [i.e., effect sizes and variations obtained post – hoc from a pilot experiment]. Table S3. PD-L1 and sTIL scores for individual patients with coefficients of variation. [file 13058_2020_1378_MOESM1_ESM.docx]

| **Supplementary Table 1. Data summary statistics** | | | | |  |  |  |  |  |  | | |  |  |
| --- | --- | --- | --- | --- | --- | --- | --- | --- | --- | --- | --- | --- | --- | --- |
| **Supp Table 1a - Cell count ratio of macrophage to helper T cell** | | | | |  |  |  |  |  |  | | |  |  |
|  | **Pre-treatment** | | | | **Post-treatment** | | | | **Combined Pre and Post** | | | | | |
| Patient ID | Mean Ratio | SD | CV | # of ROI | Mean Ratio | SD | CV | # of ROI | Mean Ratio | | | SD | CV | # of ROI |
| IRX01 | 1.44 | 0.94 | 0.65 | 11 | 0.78 | 0.52 | 0.66 | 11 | 1.11 | | | 0.81 | 0.73 | 22 |
| IRX02 | 0.72 | 0.53 | 0.74 | 11 | 0.39 | 0.23 | 0.60 | 19 | 0.51 | | | 0.40 | 0.78 | 30 |
| IRX03 | 65.45 | 103.63 | 1.58 | 11 | 0.15 | 0.09 | 0.61 | 13 | 30.08 | | | 75.99 | 2.53 | 24 |
| IRX04 | 2.22 | 1.95 | 0.87 | 9 | 3.01 | 2.97 | 0.99 | 12 | 2.67 | | | 2.56 | 0.96 | 21 |
| IRX05 | 0.10 | 0.06 | 0.61 | 12 | 0.59 | 0.75 | 1.27 | 12 | 0.34 | | | 0.58 | 1.69 | 24 |
| IRX06 | 0.18 | 0.21 | 1.19 | 12 | 0.31 | 0.21 | 0.69 | 22 | 0.26 | | | 0.22 | 0.83 | 34 |
| IRX07 | 0.49 | 0.45 | 0.92 | 13 | 1.01 | 0.67 | 0.66 | 20 | 0.81 | | | 0.64 | 0.79 | 33 |
| IRX08 | 0.67 | 0.65 | 0.97 | 13 | 0.04 | 0.05 | 1.22 | 19 | 0.30 | | | 0.51 | 1.73 | 32 |
| IRX09* | 3.50 | 3.10 | 0.89 | 9 | 1.09 | 0.96 | 0.88 | 16 | 1.96 | | | 2.27 | 1.16 | 25 |
| IRX10 | 1.16 | 1.51 | 1.30 | 13 | 0.89 | 0.69 | 0.78 | 32 | 0.97 | | | 0.99 | 1.02 | 45 |
| IRX11 | 0.67 | 0.70 | 1.05 | 13 | 0.06 | 0.07 | 1.09 | 23 | 0.28 | | | 0.51 | 1.81 | 36 |
| IRX12 | 0.23 | 0.12 | 0.52 | 12 | 0.10 | 0.08 | 0.74 | 23 | 0.15 | | | 0.11 | 0.75 | 35 |
| IRX13 | 0.38 | 0.21 | 0.56 | 12 | 0.56 | 0.46 | 0.82 | 21 | 0.50 | | | 0.39 | 0.80 | 33 |
| IRX15 | 0.13 | 0.07 | 0.51 | 7 | 0.09 | 0.06 | 0.75 | 19 | 0.10 | | | 0.07 | 0.68 | 26 |
| IRX16 | 21.91 | 31.14 | 1.42 | 8 | 0.57 | 0.62 | 1.08 | 16 | 7.69 | | | 20.02 | 2.60 | 24 |
| Note 1: **zero count for CD163+:** IRX5_post_PS17_017677_[41663,14017].im3; IRX8_post_PS17_028489_[45812,9350].im3 | | | | | | | | | | | |  |  |  |
| **zero count for CD3+:** IRX9_pre_PS17_024590_[51286,14939].im3 | | | | | | |  |  |  | | |  |  |  |
| * **Removing "IRX9_pre_PS17_024590_[51286,14939].im3"** due to zero count of CD3+ | | | | | | | |  |  | | |  |  |  |
|  |  |  |  |  |  |  |  |  |  | | |  |  |  |
| **Supp Table 1b- Cell count ratio of macrophage to T cell (any type)** | | | | | |  |  |  |  | | |  |  |  |
|  | **Pre-treatment** | | | | **Post-treatment** | | | | **Combined Pre and Post** | | | | | |
| Patient ID | Mean Ratio | SD | CV | # of ROI | Mean Ratio | SD | CV | # of ROI | Mean Ratio | | SD | | CV | # of ROI |
| IRX01 | 0.95 | 0.57 | 0.60 | 11 | 0.29 | 0.17 | 0.58 | 11 | 0.62 | | 0.53 | | 0.85 | 22 |
| IRX02 | 0.48 | 0.28 | 0.59 | 11 | 0.25 | 0.14 | 0.56 | 19 | 0.33 | | 0.23 | | 0.69 | 30 |
| IRX03 | 9.01 | 9.67 | 1.07 | 11 | 0.10 | 0.06 | 0.52 | 13 | 4.19 | | 7.82 | | 1.87 | 24 |
| IRX04 | 0.53 | 0.29 | 0.55 | 9 | 0.79 | 0.72 | 0.92 | 12 | 0.68 | | 0.58 | | 0.86 | 21 |
| IRX05 | 0.06 | 0.04 | 0.58 | 12 | 0.24 | 0.24 | 1.03 | 12 | 0.15 | | 0.19 | | 1.28 | 24 |
| IRX06 | 0.09 | 0.10 | 1.05 | 12 | 0.20 | 0.14 | 0.70 | 22 | 0.16 | | 0.13 | | 0.83 | 34 |
| IRX07 | 0.20 | 0.18 | 0.87 | 13 | 0.34 | 0.17 | 0.50 | 20 | 0.29 | | 0.18 | | 0.64 | 33 |
| IRX08 | 0.45 | 0.45 | 1.01 | 13 | 0.03 | 0.04 | 1.24 | 19 | 0.20 | | 0.35 | | 1.75 | 32 |
| IRX09 | 0.91 | 0.62 | 0.69 | 10 | 0.37 | 0.24 | 0.65 | 16 | 0.58 | | 0.50 | | 0.86 | 26 |
| IRX10 | 0.64 | 0.79 | 1.24 | 13 | 0.19 | 0.18 | 0.91 | 32 | 0.32 | | 0.48 | | 1.51 | 45 |
| IRX11 | 0.21 | 0.13 | 0.64 | 13 | 0.05 | 0.05 | 1.01 | 23 | 0.11 | | 0.12 | | 1.12 | 36 |
| IRX12 | 0.16 | 0.08 | 0.47 | 12 | 0.07 | 0.05 | 0.72 | 23 | 0.10 | | 0.07 | | 0.73 | 35 |
| IRX13 | 0.21 | 0.13 | 0.60 | 12 | 0.28 | 0.18 | 0.65 | 21 | 0.26 | | 0.17 | | 0.65 | 33 |
| IRX15 | 0.11 | 0.06 | 0.55 | 7 | 0.06 | 0.05 | 0.75 | 19 | 0.07 | | 0.05 | | 0.72 | 26 |
| IRX16 | 5.04 | 6.12 | 1.21 | 8 | 0.32 | 0.34 | 1.09 | 16 | 1.89 | | 4.08 | | 2.16 | 24 |
| Note: **zero count for CD163+**: IRX5_post_PS17_017677_[41663,14017].im3;IRX8_post_PS17_028489_[45812,9350].im3 | | | | | | | | | | |  | |  |  |
|  |  |  |  |  |  |  |  |  |  | |  | |  |  |
| **Supp Table 1c - Cell count ratio of regulatory T cell to cytotoxic T cell** | | | | |  |  |  |  |  | |  | |  |  |
|  | **Pre-treatment** | | | | **Post-treatment** | | | | **Combined Pre and Post** | | | | | |
| Patient ID | Mean Ratio | SD | CV | # of ROI | Mean Ratio | SD | CV | # of ROI | Mean Ratio | | SD | | CV | # of ROI |
| IRX01 | 0.25 | 0.13 | 0.53 | 11 | 0.17 | 0.19 | 1.09 | 11 | 0.21 | | 0.16 | | 0.77 | 22 |
| IRX02 | 0.60 | 0.31 | 0.52 | 11 | 0.12 | 0.06 | 0.50 | 19 | 0.30 | | 0.30 | | 1.02 | 30 |
| IRX03 | 0.37 | 0.37 | 1.01 | 11 | 0.09 | 0.07 | 0.85 | 13 | 0.22 | | 0.29 | | 1.34 | 24 |
| IRX04 | 10.49 | 11.38 | 1.09 | 9 | 1.23 | 0.99 | 0.80 | 12 | 5.20 | | 8.62 | | 1.66 | 21 |
| IRX05 | 1.34 | 2.10 | 1.57 | 12 | 0.30 | 0.31 | 1.03 | 12 | 0.82 | | 1.56 | | 1.90 | 24 |
| IRX06 | 0.79 | 0.41 | 0.52 | 12 | 0.10 | 0.06 | 0.66 | 22 | 0.34 | | 0.42 | | 1.22 | 34 |
| IRX07 | 0.67 | 0.59 | 0.87 | 13 | 0.07 | 0.05 | 0.82 | 20 | 0.30 | | 0.47 | | 1.54 | 33 |
| IRX08 | 1.35 | 2.11 | 1.57 | 13 | 1.00 | 0.56 | 0.56 | 19 | 1.14 | | 1.39 | | 1.22 | 32 |
| IRX09* | 1.69 | 2.08 | 1.23 | 9 | 0.28 | 0.30 | 1.08 | 16 | 0.79 | | 1.41 | | 1.78 | 25 |
| IRX10 | 0.62 | 0.43 | 0.69 | 13 | 0.21 | 0.12 | 0.55 | 32 | 0.33 | | 0.31 | | 0.94 | 45 |
| IRX11 | 0.51 | 0.32 | 0.62 | 13 | 0.36 | 0.22 | 0.62 | 23 | 0.41 | | 0.27 | | 0.65 | 36 |
| IRX12 | 0.40 | 0.38 | 0.94 | 12 | 0.05 | 0.03 | 0.67 | 23 | 0.17 | | 0.28 | | 1.62 | 35 |
| IRX13 | 0.08 | 0.05 | 0.61 | 12 | 0.14 | 0.09 | 0.64 | 21 | 0.12 | | 0.08 | | 0.69 | 33 |
| IRX15 | 0.21 | 0.04 | 0.18 | 7 | 0.11 | 0.06 | 0.54 | 19 | 0.14 | | 0.07 | | 0.50 | 26 |
| IRX16 | 0.33 | 0.42 | 1.26 | 8 | 0.07 | 0.07 | 0.98 | 16 | 0.16 | | 0.27 | | 1.72 | 24 |
| Note 1: **zero count for CD8+**: IRX9_pre_PS17_024590_[51458,12296].im3 | | | | | | | | | | |  | |  |  |
| **zero count for FOXP3+**: IRX3_pre_PS17_011106_[41569,16196].im3; IRX2_post_PS17_019002_[44859,9434].im3; IRX6_post_PS17_023858_[53969,11106].im3; IRX7_post_PS17_23351_[50940,18076].im3; IRX16_post_PS18_020840_[44632,18686].im3; IRX16_post_PS18_020840_[52477,19213].im3 | | | | | | | | | | | | | | |
|  |  |  |  |  |  |  |  |  |  |  |  |  |  |  |
|  |  |  |  |  |  |  |  |  |  |  |  |  |  |  |
| * **Removing "IRX9_pre_PS17_024590_[51458,12296].im3"** due to zero count of CD8+ | | | | | | | |  |  |  | | |  |  |
|  |  |  |  |  |  |  |  |  |  |  | | |  |  |
| **Supp Table 1d - Cell count ratio of cytotoxic T cell to helper T cell** | | | | |  |  |  |  |  |  | | |  |  |
|  | **Pre-treatment** | | | | **Post-treatment** | | | | **Combined Pre and Post** | | | | | |
| Patient ID | Mean Ratio | SD | CV | # of ROI | Mean Ratio | SD | CV | # of ROI | Mean Ratio | | SD | | CV | # of ROI |
| IRX01 | 0.39 | 0.13 | 0.32 | 11 | 1.39 | 0.40 | 0.29 | 11 | 0.89 | | 0.58 | | 0.66 | 22 |
| IRX02 | 0.31 | 0.19 | 0.61 | 11 | 0.47 | 0.10 | 0.21 | 19 | 0.41 | | 0.16 | | 0.38 | 30 |
| IRX03 | 3.19 | 1.88 | 0.59 | 11 | 0.32 | 0.16 | 0.50 | 13 | 1.64 | | 1.92 | | 1.17 | 24 |
| IRX04 | 0.40 | 0.33 | 0.82 | 9 | 1.04 | 0.75 | 0.72 | 12 | 0.77 | | 0.68 | | 0.88 | 21 |
| IRX05 | 0.23 | 0.10 | 0.46 | 12 | 1.18 | 0.96 | 0.81 | 12 | 0.70 | | 0.82 | | 1.17 | 24 |
| IRX06 | 0.41 | 0.13 | 0.31 | 12 | 0.52 | 0.15 | 0.28 | 22 | 0.48 | | 0.15 | | 0.31 | 34 |
| IRX07 | 0.82 | 0.25 | 0.31 | 13 | 1.70 | 0.80 | 0.47 | 20 | 1.35 | | 0.77 | | 0.57 | 33 |
| IRX08 | 0.23 | 0.22 | 0.98 | 13 | 0.19 | 0.13 | 0.68 | 19 | 0.20 | | 0.17 | | 0.84 | 32 |
| IRX09 | 1.72 | 2.86 | 1.66 | 9 | 1.44 | 0.85 | 0.59 | 16 | 1.54 | | 1.79 | | 1.16 | 25 |
| IRX10 | 0.48 | 0.23 | 0.49 | 13 | 3.73 | 3.35 | 0.90 | 32 | 2.79 | | 3.18 | | 1.14 | 45 |
| IRX11 | 1.03 | 0.70 | 0.68 | 13 | 0.21 | 0.07 | 0.32 | 23 | 0.50 | | 0.58 | | 1.14 | 36 |
| IRX12 | 0.31 | 0.08 | 0.28 | 12 | 0.48 | 0.15 | 0.30 | 23 | 0.42 | | 0.15 | | 0.36 | 35 |
| IRX13 | 0.79 | 0.24 | 0.30 | 12 | 0.76 | 0.34 | 0.45 | 21 | 0.77 | | 0.30 | | 0.39 | 33 |
| IRX15 | 0.19 | 0.04 | 0.19 | 7 | 0.36 | 0.32 | 0.88 | 19 | 0.31 | | 0.28 | | 0.89 | 26 |
| IRX16 | 1.63 | 0.38 | 0.23 | 8 | 0.72 | 0.33 | 0.46 | 16 | 1.03 | | 0.55 | | 0.54 | 24 |
| Note 1: **zero count for CD8+:** IRX9_pre_PS17_024590_[51458,12296].im3 | | | | | | | | | | |  | |  |  |
| **zero count for CD3+:** IRX9_pre_PS17_024590_[51286,14939].im3 | | | | | | |  |  |  | |  | |  |  |
| * **Removing "IRX9_pre_PS17_024590_[51286,14939].im3"** due to zero count of CD3+ | | | | | | | |  |  | |  | |  |  |

| **Supplementary Table 2.** PD-L1 and sTIL scores for individual patients with coefficients of variation | | | | | | | | | | | | |
| --- | --- | --- | --- | --- | --- | --- | --- | --- | --- | --- | --- | --- |
| Supp Table 2a- PD-L1mIF (i.e., PD-L1>2.6 on T-cell, any type, or macrophage). | | | | | | | | | | | | |
|  | Pre-treatment | | | | Post-treatment | | | | Cimbined Pre and Post | | | |
| Patient ID | Mean Density*  (count/pixel) | SD* | CV | # of ROI | Mean Density*  (count/pixel) | SD* | CV | # of ROI | Mean Density*  (count/pixel) | SD* | CV | # of ROI |
| IRX01 | 0.07 | 0.08 | 1.13 | 11 | 0.13 | 0.10 | 0.78 | 11 | 0.10 | 0.09 | 0.94 | 22 |
| IRX02 | 1.04 | 0.83 | 0.80 | 11 | 0.87 | 0.36 | 0.41 | 19 | 0.93 | 0.57 | 0.61 | 30 |
| IRX03 | 0.07 | 0.06 | 0.90 | 11 | 0.35 | 0.25 | 0.73 | 13 | 0.22 | 0.24 | 1.08 | 24 |
| IRX04 | 0.24 | 0.17 | 0.72 | 9 | 0.68 | 0.58 | 0.85 | 12 | 0.49 | 0.49 | 1.01 | 21 |
| IRX05 | 0.18 | 0.17 | 0.97 | 12 | 0.09 | 0.10 | 1.09 | 12 | 0.14 | 0.15 | 1.07 | 24 |
| IRX06 | 0.01 | 0.01 | 0.95 | 8 | 0.40 | 0.25 | 0.63 | 22 | 0.30 | 0.28 | 0.94 | 30 |
| IRX07 | 0.54 | 0.51 | 0.94 | 13 | 0.64 | 0.41 | 0.64 | 20 | 0.60 | 0.44 | 0.74 | 33 |
| IRX08 | 0.97 | 0.86 | 0.89 | 13 | 1.30 | 0.87 | 0.67 | 19 | 1.17 | 0.87 | 0.75 | 32 |
| IRX09 | 0.06 | 0.06 | 0.95 | 10 | 0.23 | 0.25 | 1.08 | 16 | 0.16 | 0.21 | 1.30 | 26 |
| IRX10 | 0.38 | 0.32 | 0.85 | 13 | 1.33 | 0.84 | 0.63 | 32 | 1.06 | 0.84 | 0.80 | 45 |
| IRX11 | 0.22 | 0.28 | 1.29 | 13 | 0.65 | 0.36 | 0.55 | 23 | 0.49 | 0.39 | 0.79 | 36 |
| IRX12 | 1.81 | 0.55 | 0.30 | 12 | 1.99 | 0.74 | 0.37 | 23 | 1.93 | 0.68 | 0.35 | 35 |
| IRX13 | 0.06 | 0.06 | 0.90 | 12 | 0.98 | 0.37 | 0.38 | 21 | 0.65 | 0.54 | 0.83 | 33 |
| IRX15 | n/a | n/a | n/a | 0 | 0.76 | 0.35 | 0.46 | 19 | 0.76 | 0.35 | 0.46 | 19 |
| IRX16 | 0.03 | 0.03 | 0.88 | 7 | 0.06 | 0.06 | 0.95 | 16 | 0.05 | 0.05 | 1.00 | 23 |
|  |  |  |  |  |  |  |  |  |  |  |  |  |
| Supp Table 2b - sTILmIF (i.e., T-cell, any type, in stroma). | | | | | | | | | | | | |
|  | Pre-treatment | | | | Post-treatment | | | | Combined Pre and Post | | | |
| Patient ID | Mean Density*  (count/pixel) | SD* | CV | # of ROI | Mean Density*  (count/pixel) | SD* | CV | # of ROI | Mean Density*  (count/pixel) | SD* | CV | # of ROI |
| IRX01 | 0.48 | 0.26 | 0.55 | 11 | 0.59 | 0.24 | 0.41 | 11 | 0.54 | 0.25 | 0.47 | 22 |
| IRX02 | 1.35 | 0.72 | 0.53 | 11 | 1.53 | 0.59 | 0.38 | 19 | 1.46 | 0.63 | 0.43 | 30 |
| IRX03 | 0.13 | 0.19 | 1.44 | 11 | 1.25 | 0.65 | 0.52 | 13 | 0.74 | 0.75 | 1.01 | 24 |
| IRX04 | 0.23 | 0.13 | 0.56 | 9 | 0.74 | 0.65 | 0.88 | 12 | 0.52 | 0.55 | 1.06 | 21 |
| IRX05 | 0.88 | 0.91 | 1.03 | 12 | 0.34 | 0.17 | 0.50 | 12 | 0.61 | 0.70 | 1.15 | 24 |
| IRX06 | 0.84 | 0.38 | 0.45 | 12 | 0.94 | 0.40 | 0.43 | 22 | 0.90 | 0.39 | 0.43 | 34 |
| IRX07 | 1.18 | 1.03 | 0.87 | 13 | 1.24 | 0.73 | 0.59 | 20 | 1.22 | 0.85 | 0.70 | 33 |
| IRX08 | 1.28 | 1.03 | 0.80 | 13 | 1.68 | 0.94 | 0.56 | 19 | 1.52 | 0.98 | 0.64 | 32 |
| IRX09 | 0.06 | 0.03 | 0.50 | 10 | 0.27 | 0.23 | 0.86 | 16 | 0.19 | 0.21 | 1.09 | 26 |
| IRX10 | 0.41 | 0.36 | 0.89 | 13 | 1.48 | 0.87 | 0.58 | 32 | 1.17 | 0.90 | 0.77 | 45 |
| IRX11 | 0.35 | 0.35 | 0.98 | 13 | 1.59 | 0.54 | 0.34 | 23 | 1.15 | 0.77 | 0.67 | 36 |
| IRX12 | 2.21 | 0.69 | 0.31 | 12 | 3.52 | 0.90 | 0.26 | 23 | 3.07 | 1.04 | 0.34 | 35 |
| IRX13 | 1.69 | 0.89 | 0.53 | 12 | 1.77 | 0.86 | 0.48 | 21 | 1.74 | 0.86 | 0.49 | 33 |
| IRX15 | 1.26 | 0.34 | 0.27 | 7 | 1.14 | 0.50 | 0.44 | 19 | 1.17 | 0.46 | 0.39 | 26 |
| IRX16 | 0.38 | 0.32 | 0.84 | 8 | 0.99 | 0.62 | 0.63 | 16 | 0.79 | 0.61 | 0.78 | 24 |
|  |  |  |  |  |  |  |  |  |  |  |  |  |
| Supp Table 2c - T-cell (any type) in tumor | | | | | | | | | | | | |
|  | Pre-treatment | | | | Post-treatment | | | | Combined Pre and Post | | | |
| Patient ID | Mean Density*  (count/pixel) | SD* | CV | # of ROI | Mean Density*  (count/pixel) | SD* |  | # of ROI | Mean Density*  (count/pixel) | SD* | CV | # of ROI |
| IRX01 | 0.10 | 0.10 | 0.91 | 11 | 0.04 | 0.04 | 1.07 | 10 | 0.07 | 0.08 | 1.10 | 21 |
| IRX02 | 0.37 | 0.24 | 0.66 | 9 | 0.18 | 0.12 | 0.68 | 19 | 0.24 | 0.19 | 0.79 | 28 |
| IRX03 | 0.01 | 0.00 | 0.21 | 4 | 0.04 | 0.03 | 0.60 | 10 | 0.03 | 0.03 | 0.83 | 14 |
| IRX04 | 0.06 | 0.10 | 1.52 | 7 | 0.03 | 0.02 | 0.84 | 9 | 0.04 | 0.07 | 1.57 | 16 |
| IRX05 | 0.03 | 0.04 | 1.26 | 10 | 0.03 | 0.04 | 1.39 | 8 | 0.03 | 0.04 | 1.28 | 18 |
| IRX06 | 0.16 | 0.07 | 0.45 | 12 | 0.04 | 0.02 | 0.66 | 22 | 0.08 | 0.08 | 0.96 | 34 |
| IRX07 | 0.29 | 0.29 | 0.99 | 13 | 0.46 | 0.46 | 1.01 | 20 | 0.39 | 0.40 | 1.04 | 33 |
| IRX08 | 0.14 | 0.18 | 1.30 | 13 | 0.08 | 0.06 | 0.67 | 19 | 0.11 | 0.13 | 1.17 | 32 |
| IRX09 | 0.01 | 0.01 | 0.90 | 5 | 0.05 | 0.07 | 1.39 | 13 | 0.04 | 0.06 | 1.55 | 18 |
| IRX10 | 0.04 | 0.04 | 0.94 | 6 | 0.08 | 0.05 | 0.62 | 24 | 0.07 | 0.05 | 0.70 | 30 |
| IRX11 | 0.09 | 0.17 | 1.77 | 11 | 0.37 | 0.28 | 0.76 | 23 | 0.28 | 0.28 | 1.00 | 34 |
| IRX12 | 0.61 | 0.29 | 0.48 | 12 | 0.43 | 0.44 | 1.02 | 23 | 0.49 | 0.40 | 0.81 | 35 |
| IRX13 | 0.38 | 0.18 | 0.48 | 12 | 0.21 | 0.17 | 0.81 | 21 | 0.27 | 0.19 | 0.71 | 33 |
| IRX15 | 0.08 | 0.06 | 0.76 | 5 | 0.11 | 0.08 | 0.72 | 19 | 0.10 | 0.08 | 0.74 | 24 |
| IRX16 | 0.06 | 0.12 | 2.02 | 7 | 0.07 | 0.09 | 1.28 | 11 | 0.07 | 0.10 | 1.50 | 18 |
| *x10^-3^ |  |  |  |  |  |  |  |  |  |  |  |  |

**Supplementary Table 3.** Power analysis using Monte Carlo simulation approach (n=1,000 simulations), based on the generalized linear mixed-effects model and the observed data structure [i.e., effect sizes and variations obtained post – hoc from a pilot experiment]

Supp Table 3a- The power curve with patient sample size, holding ROI size fixed based on observed data structure for PD-L1mIF (i.e., PD-L1>2.6 on T-cell, any type, or macrophage).

| Total number of ROI | Number of patient | Power | Lower 95% CI | Upper 95% CI |
| --- | --- | --- | --- | --- |
| 121 | 5 | 0.54 | 0.51 | 0.58 |
| 151 | 6 | 0.60 | 0.57 | 0.63 |
| 184 | 7 | 0.68 | 0.65 | 0.71 |
| 216 | 8 | 0.73 | 0.70 | 0.76 |
| 242 | 9 | 0.79 | 0.77 | 0.82 |
| 287 | 10 | 0.82 | 0.79 | 0.84 |
| 323 | 11 | 0.85 | 0.82 | 0.87 |
| 358 | 12 | 0.89 | 0.86 | 0.90 |
| 391 | 13 | 0.91 | 0.89 | 0.92 |
| 410 | 14 | 0.91 | 0.89 | 0.92 |
| 433 | 15 | 0.93 | 0.91 | 0.94 |

Supp Table 3b- The power curve with patient sample size, holding ROI size fixed based on observed data structure for sTILmIF (i.e., stromal T-cells, any type).

| Total number of ROI | Number of patient | Power | Lower 95% CI | Upper 95% CI |
| --- | --- | --- | --- | --- |
| 121 | 5 | 0.46 | 0.42 | 0.49 |
| 155 | 6 | 0.53 | 0.49 | 0.56 |
| 188 | 7 | 0.58 | 0.55 | 0.61 |
| 220 | 8 | 0.65 | 0.62 | 0.68 |
| 246 | 9 | 0.70 | 0.67 | 0.73 |
| 291 | 10 | 0.75 | 0.73 | 0.78 |
| 327 | 11 | 0.78 | 0.75 | 0.80 |
| 362 | 12 | 0.81 | 0.79 | 0.83 |
| 395 | 13 | 0.85 | 0.83 | 0.87 |
| 421 | 14 | 0.87 | 0.85 | 0.89 |
| 445 | 15 | 0.89 | 0.87 | 0.91 |
