# Supplementary material for: Multiplex immunofluorescence to measure dynamic changes in tumor-infiltrating lymphocytes and PD-L1 in early-stage breast cancer
Source: Breast Cancer Res. 2021 Jan 7;23:2. doi: 10.1186/s13058-020-01378-4 (PMC7788790; doi:10.1186/s13058-020-01378-4)
Supplement: Supplementary file 2 — Additional file 2: Figure S1. Receiver operating characteristic curve for determining PD-L1 threshold. (A) Example images of high powered (20x) ROIs, InForm pathology view (showing only PD-L1 expression by mIF) counterstained with DAPI. Green is used here to label random cells that could be visually classified as PD-L1 positive versus negative by the reading pathologist, and used to ascertain an appropriate QIF cutoff for PD-L1 positivity. (B) Histogram of the distribution average per-cell QIF PD-L1 levels of 55,108 cells pooled from 24 ROI across 4 patients). (C) ROC curve illustrating sensitivity and specificity for given PD-L1 QIF thresholds. A threshold of ≥2.6 was selected, corresponding with sensitivity of 91%, specificity of 99%, AUC of 0.97, and accuracy of 95%. ROI: region of interest; PD-L1: programmed death ligand 1; mIF: multispectral immunofluorescence; DAPI: 4′,6-diamidino-2-phenylindole; QIF: quantitative immunofluorescence; ROC: receiver operating characteristic; AUC: area under the curve. [file 13058_2020_1378_MOESM2_ESM.pptx]

## Slide 1
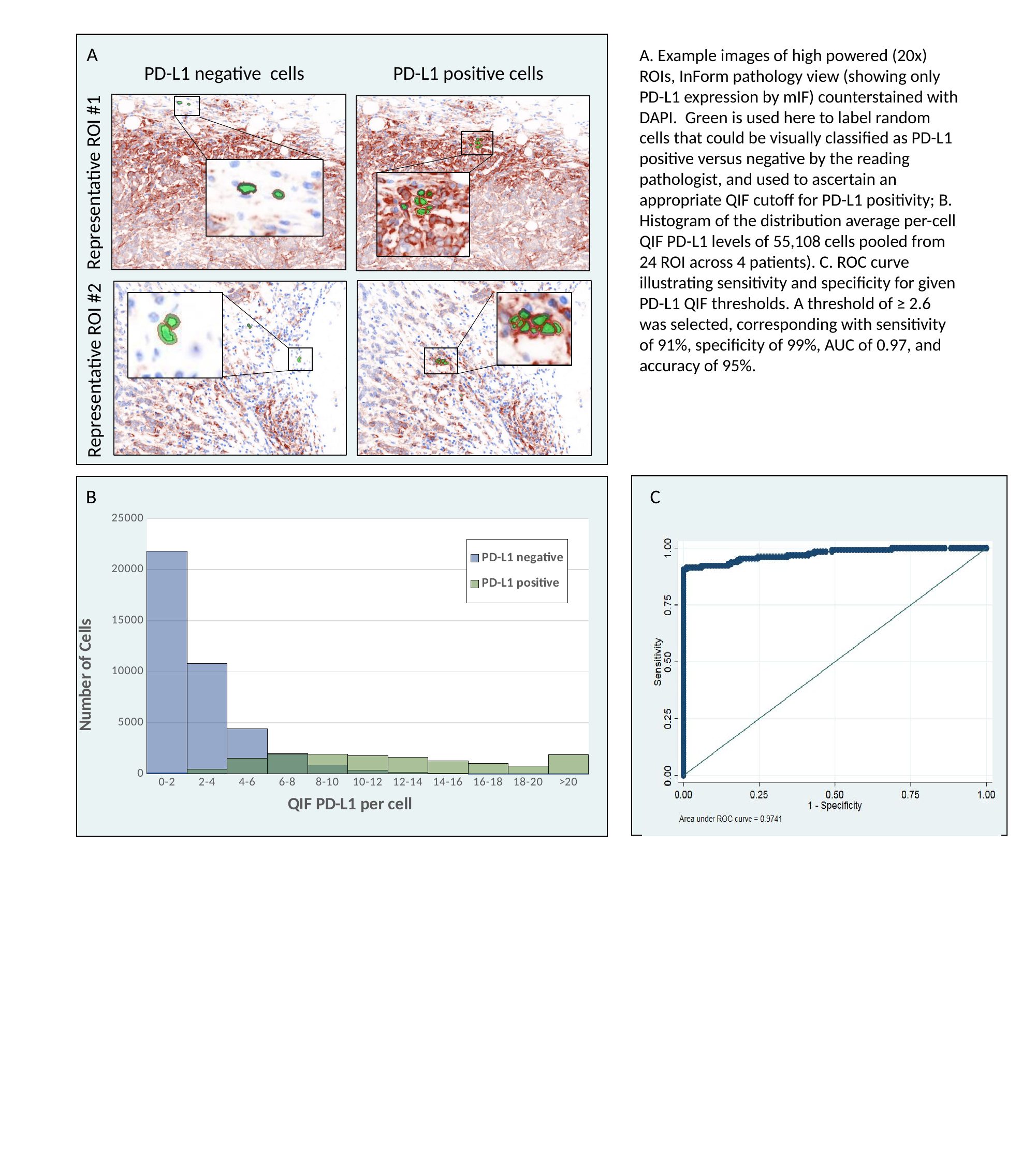

A
A. Example images of high powered (20x) ROIs, InForm pathology view (showing only PD-L1 expression by mIF) counterstained with DAPI. Green is used here to label random cells that could be visually classified as PD-L1 positive versus negative by the reading pathologist, and used to ascertain an appropriate QIF cutoff for PD-L1 positivity; B. Histogram of the distribution average per-cell QIF PD-L1 levels of 55,108 cells pooled from 24 ROI across 4 patients). C. ROC curve illustrating sensitivity and specificity for given PD-L1 QIF thresholds. A threshold of ≥ 2.6 was selected, corresponding with sensitivity of 91%, specificity of 99%, AUC of 0.97, and accuracy of 95%.
PD-L1 negative cells
PD-L1 positive cells
Representative ROI #1
Representative ROI #2
C
B
B
### Chart
| Category | | PD-L1 negative | PD-L1 positive |
|---|---|---|---|
| 0-2 | 126.0 | 21796.0 | 9.0 |
| 2-4 | 60.0 | 10816.0 | 453.0 |
| 4-6 | 55.0 | 4427.0 | 1532.0 |
| 6-8 | 29.0 | 1988.0 | 1939.0 |
| 8-10 | 18.0 | 862.0 | 1949.0 |
| 10-12 | 11.0 | 354.0 | 1789.0 |
| 12-14 | 14.0 | 138.0 | 1606.0 |
| 14-16 | 11.0 | 64.0 | 1299.0 |
| 16-18 | 7.0 | 27.0 | 1046.0 |
| 18-20 | 3.0 | 7.0 | 776.0 |
| >20 | 8.0 | 12.0 | 1887.0 |
