# Supplementary figures and images for: Multiplex immunofluorescence to measure dynamic changes in tumor-infiltrating lymphocytes and PD-L1 in early-stage breast cancer
Source: Breast Cancer Res. 2021 Jan 7;23:2. doi: 10.1186/s13058-020-01378-4 (PMC7788790; doi:10.1186/s13058-020-01378-4)

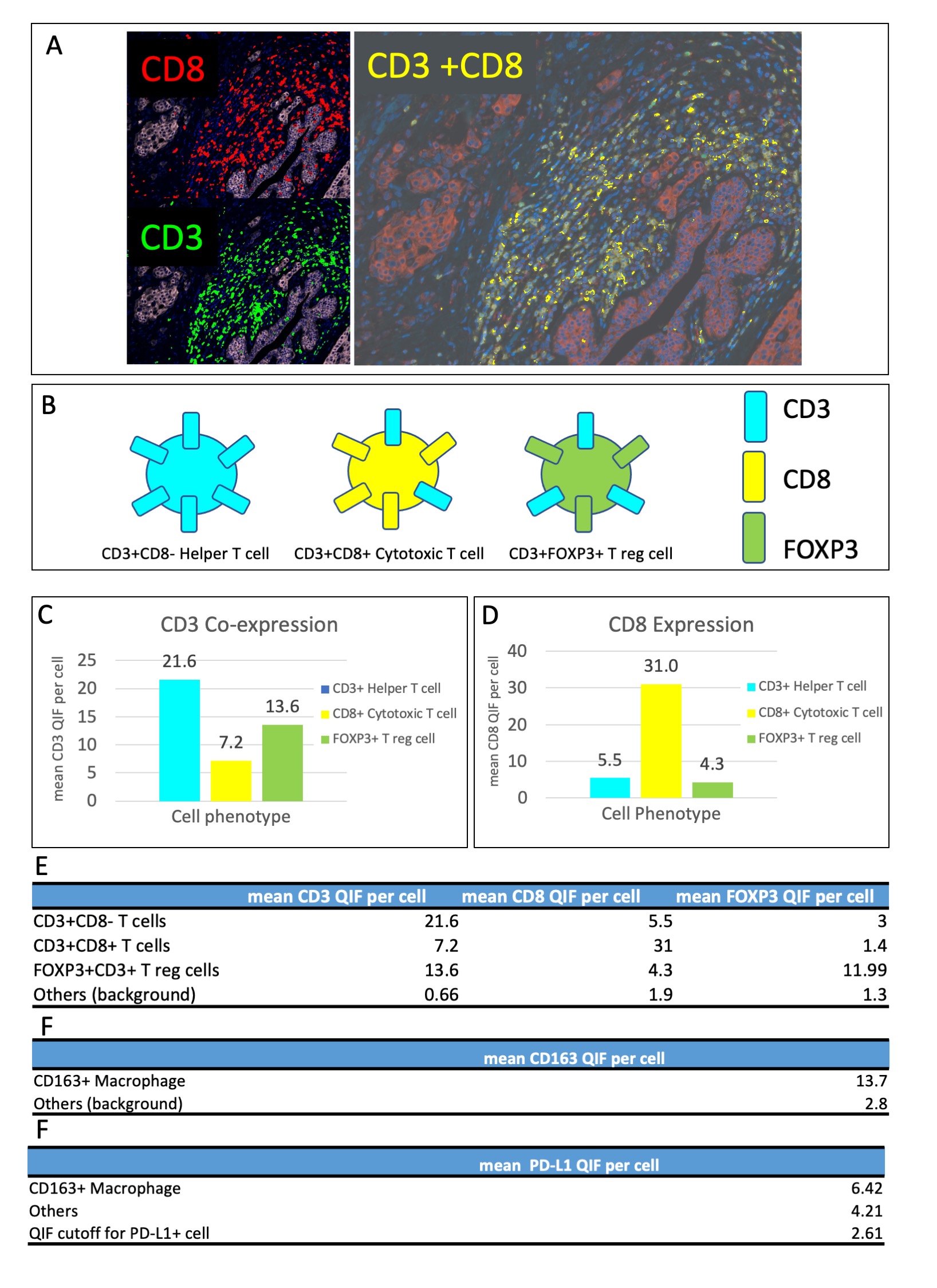

Supplement: Supplementary file 3 — Additional file 3: Figure S2. Example of mIF staining and illustration of QIF levels across phenotypes using machine-learning based (InForm) phenotyping method. PD-L1: programmed death ligand 1; DAPI: 4′,6-diamidino-2-phenylindole; CK: cytokeratin; FOXP3. (A) mIF images showing CK+ tumor nests with CD8 expression (red), CD3 expression (green), and yellow indicating co-expression; (B) expected expression patterns of helper T cells, cytotoxic T cells, and regulatory T cells; (C-D) Mean quantitative immunofluorescence of CD3 and CD8 for each of the phenotypes; (E-G) Comparison of mean QIF expression patterns for various phenotypes macrophage CD163 expression to other cells. CK: cytokeratin; FOXP3: forkhead box P3; reg: regulatory; QIF: quantitative immunofluorescence. [file 13058_2020_1378_MOESM3_ESM.jpeg]

## Slide 1
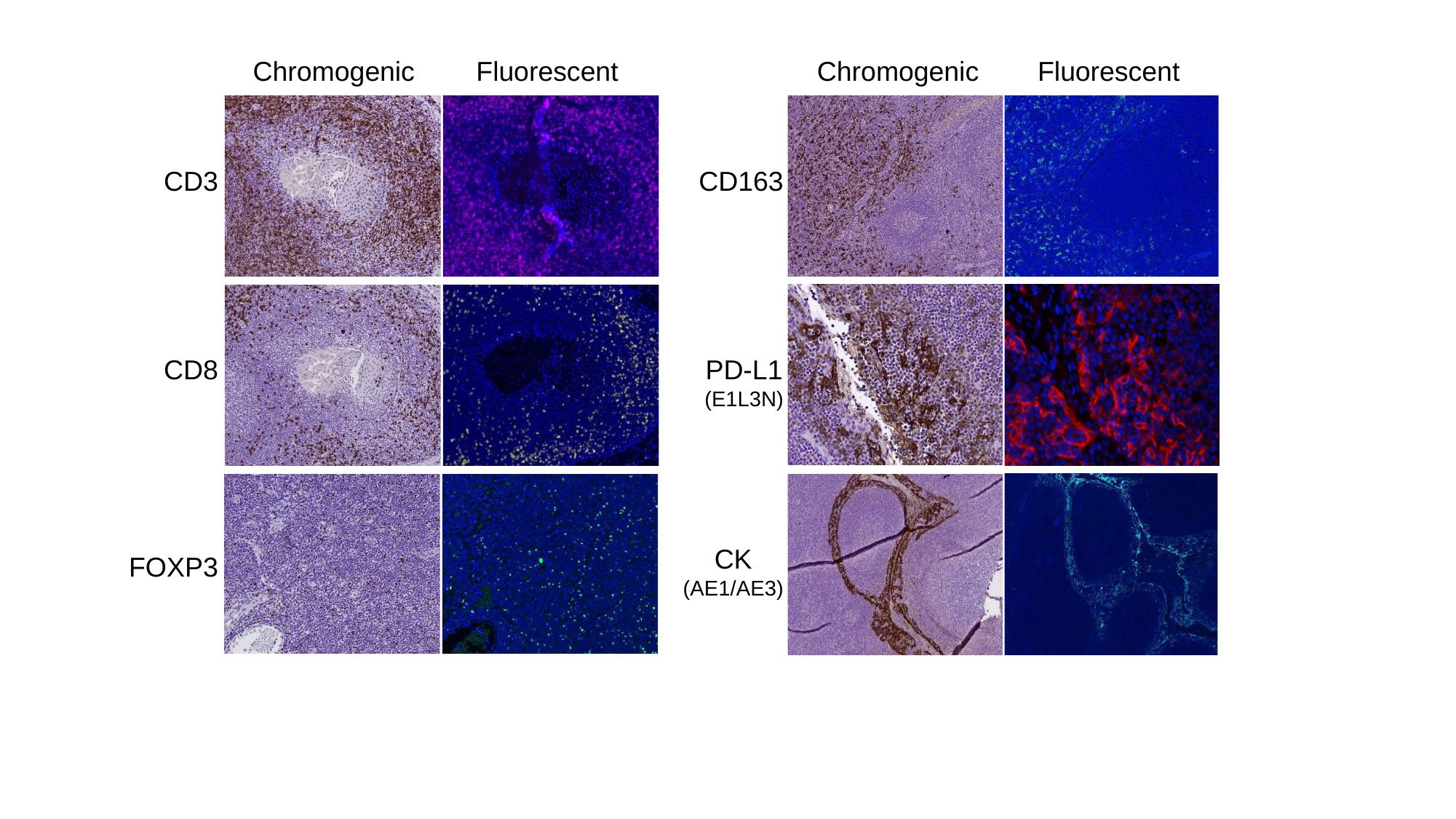

Chromogenic
Fluorescent
Chromogenic
Fluorescent
CD3
CD163
CD8
PD-L1
(E1L3N)
CK
(AE1/AE3)
FOXP3

Supplement: Supplementary file 4 — Additional file 4: Figure S3. mIF antibody validation. Antibody validation was performed using conventional/standard chromogenic stain on human FFPE tonsil tissue. TSA-Opal fluorescent stain was performed on the adjacent slides. Images were acquired with a Vectra 3 Automated Quantitative Pathology Imaging system. FOXP3: forkhead box P3; CK: cytokeratin; PD-L1: programmed death ligand 1. [file 13058_2020_1378_MOESM4_ESM.pptx]

## Slide 1
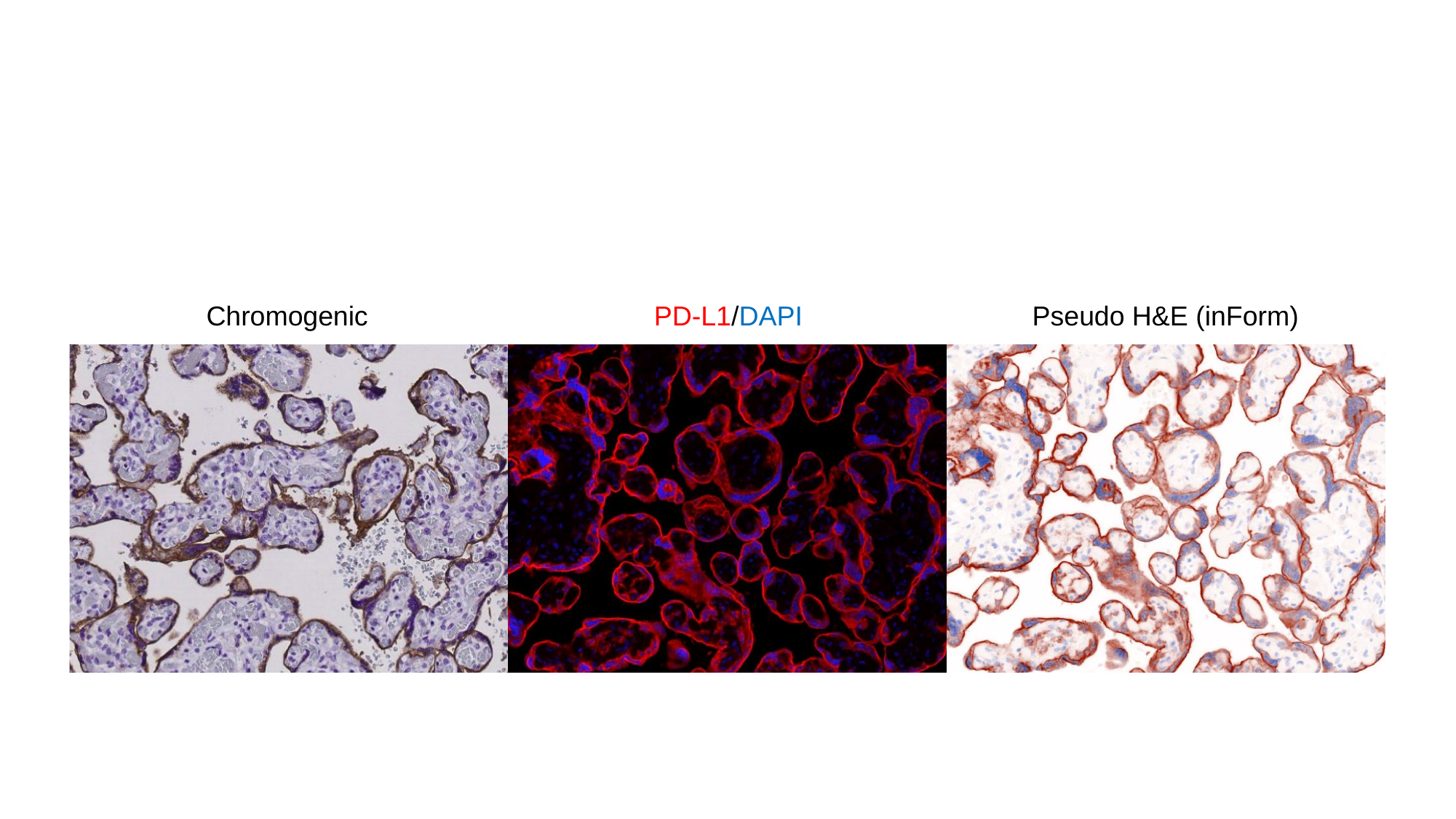

Chromogenic
PD-L1/DAPI
Pseudo H&E (inForm)

Supplement: Supplementary file 5 — Additional file 5: Figure S4. mIF antibody validation, PD-L1 staining. Antibody validation (aPD-L1; clone E1L3N) was performed using conventional/standard chromogenic stain on human FFPE placenta tissue. TSA-Opal fluorescent stain was performed on the adjacent slides. Images were taken with a Vectra 3 Automated Quantitative Pathology Imaging system. PD-L1: programmed death ligand 1; H&E: Hematoxylin and eosin. [file 13058_2020_1378_MOESM5_ESM.pptx]
